# Synthesis of a Phosphoethanolamine Cellulose Mimetic and Evaluation of Its Unanticipated Biofilm Modulating Properties

**DOI:** 10.1021/acsinfecdis.4c00267

**Published:** 2024-08-06

**Authors:** C. Elizabeth Adams, Sabrina K. Spicer, Jennifer A. Gaddy, Steven D. Townsend

**Affiliations:** † Department of Chemistry, Vanderbilt University, Nashville, Tennessee 37235, United States; ‡ Department of Medicine, Vanderbilt University Medical Center, Nashville, Tennessee 37232, United States; § Department of Veterans Affairs, Tennessee Valley Healthcare Systems, Nashville, Tennessee 37212, United States; ∥ Department of Pathology, Microbiology and Immunology, Vanderbilt University Medical Center, Nashville, Tennessee 37232, United States

**Keywords:** phosphoethanolamine cellulose, biofilms, Escherichia coli, glycopolymers

## Abstract

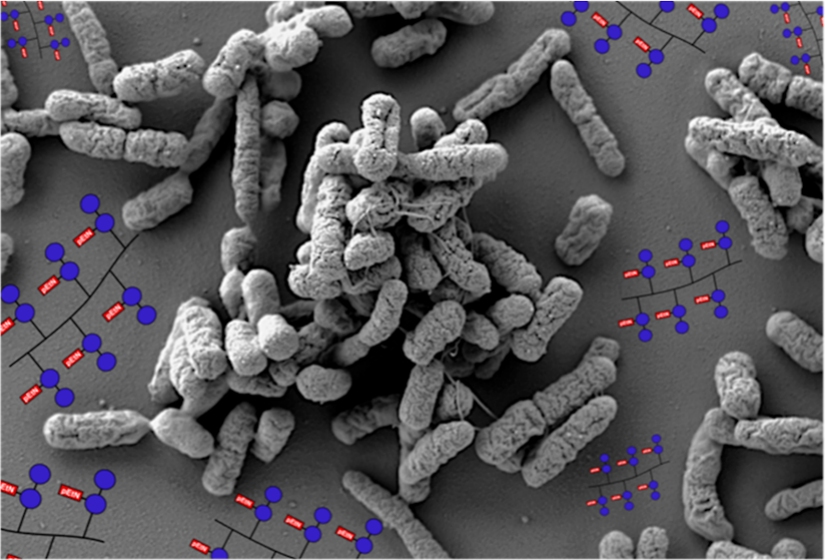

When coordinating and adhering to a surface, microorganisms produce a biofilm matrix consisting of extracellular DNA, lipids, proteins, and polysaccharides that are intrinsic to the survival of bacterial communities. Indeed, bacteria produce a variety of structurally diverse polysaccharides that play integral roles in the emergence and maintenance of biofilms by providing structural rigidity, adhesion, and protection from environmental stressors. While the roles that polysaccharides play in biofilm dynamics have been described for several bacterial species, the difficulty in isolating homogeneous material has resulted in few structures being elucidated. Recently, Cegelski and co-workers discovered that uropathogenic *Escherichia coli* (UPEC) secrete a chemically modified cellulose called phosphoethanolamine cellulose (pEtN cellulose) that plays a vital role in biofilm assembly. However, limited chemical tools exist to further examine the functional role of this polysaccharide across bacterial species. To address this critical need, we hypothesized that we could design and synthesize an unnatural glycopolymer to mimic the structure of pEtN cellulose. Herein, we describe the synthesis and evaluation of a pEtN cellulose glycomimetic which was generated using ring-opening metathesis polymerization. Surprisingly, the synthetic polymers behave counter to native pEtN cellulose in that the synthetic polymers repress biofilm formation in *E. coli* laboratory strain 11775T and UPEC strain 700415 with longer glycopolymers displaying greater repression. To evaluate the mechanism of action, changes in biofilm and cell morphology were visualized using high resolution field-emission gun scanning electron microscopy which further revealed changes in cell surface appendages. Our results suggest synthetic pEtN cellulose glycopolymers act as an antiadhesive and inhibit biofilm formation across *E. coli* strains, highlighting a potential new inroad to the development of bioinspired, biofilm-modulating materials.

To cooperate and coordinate within a community, microorganisms form aggregates of cells encased in a rigid, yet dynamic extracellular matrix that is commonly referred to as a biofilm.^1^ Bacterial biofilm formation is an ordered cycle that can be summarized into five stages (Figure 1). The first stage encompasses reversible attachment, wherein the microbial cell adheres to a biotic or abiotic surface via van der Waals forces. This is followed by a period of coordination where cells irreversibly attach to the surface via hydrophilic/hydrophobic interactions facilitated by appendages and macromolecules such as exopolysaccharide, lipopolysaccharide, flagella, and pili. The third stage involves the formation of microcolonies where layers of cells are accumulated. Stage four features maturation of the biofilm, specifically formation of its three-dimensional structure, including development of pores and channels to enable active transport of nutrients, signaling molecules, and waste. The final stage of the biofilm life cycle is dispersal—bacterial cells detach due to either intrinsic or extrinsic factors such as nutrient levels, pH, or temperature. Biofilm formation is key to conferring bacterial pathogenicity and viability; specifically the biofilm matrix offers a physical layer separating the bacterial cell from the environment and ultimately serves to increase bacterial survival from challenges such as antimicrobial agents or other bacterial species.^2^ Biofilms also help bacteria evade host immune responses including phagocytosis by innate immune cells, and protect from various environmental stressors such as desiccation or osmotic pressure.^3^ Biofilms aid in adhesion and survival and, therefore, are an important virulence factor among pathogenic bacteria, increasing the ability of bacteria to adapt to new niches and to cause a broad spectrum of disease.^3^

**Figure 1:**
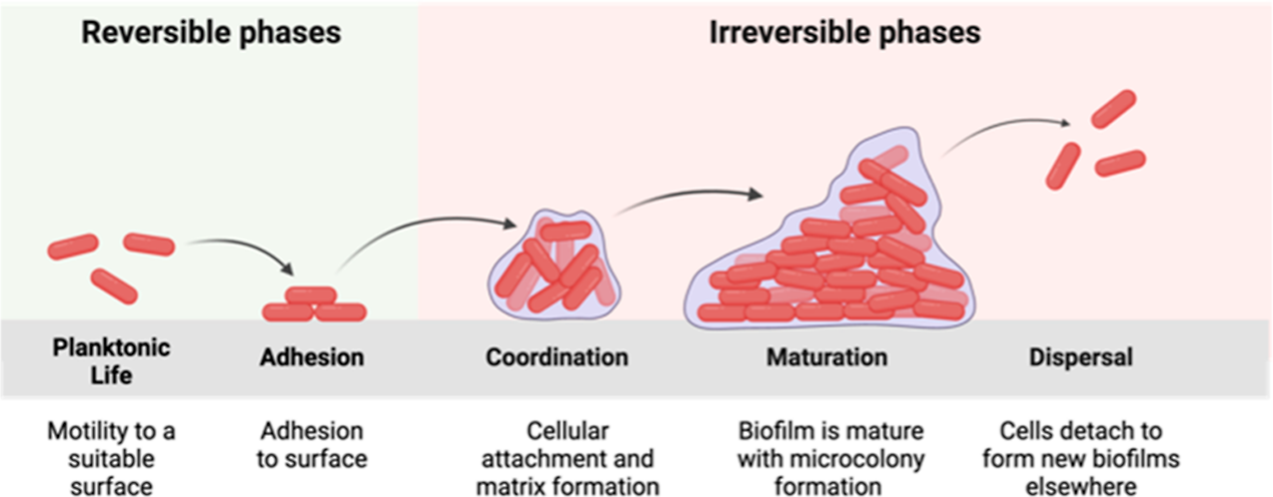
Stages of biofilm formation and maturation.

*Escherichia coli* is one bacterium most frequently associated with biofilm-related infections.^1^ This behavior is easily observed in uropathogenic *E. coli* (UPEC), a microorganism associated with chronic and persistent inflammation leading to complex and recurrent urinary tract infections (UTIs). Indeed in recent years 90% of UTIs are attributable to UPEC strains.^4,5^ Globally, UTIs are the most common infection among humans and successful establishment of infection requires bacterial adhesion to host cells.^3,6^ The establishment of a UPEC biofilm progresses from reversible to irreversible attachment—indicating a mature biofilm.^1,7^ Initiating reversible attachment requires coordination of bacteria to a suitable surface for adhesion. In *E. coli* this is facilitated by expression of flagella which increase cellular motility and overcome electrostatic forces between cells and the surface. Following reversible attachment *E. coli* cells assess environmental conditions and transition to irreversible attachment through the use of fimbriae such as conjugative pili, curli fimbriae, and type 1 pili.^1,3^ Once irreversible attachment is initiated, matrix production begins.

While mainly composed of water, the biofilm matrix houses polysaccharides, proteins, nucleic acids, lipids/phospholipids, nutrients, and metabolites.^8^ Due to the complexity of biofilm matrices, detailed structural characterization of the extracellular polymeric matrix is minimal. Of the key elements that have been discovered within biofilm matrices across microorganisms, cellulose performs vital protective, architectural, and regulatory functions during formation of a mature biofilm.^8^ Indeed, cellulose is one of the most abundant polysaccharides in nature, providing important structural rigidity to the matrix and is an integral component of biofilms across Gram-negative and Gram-positive bacteria.^2,8^

Detailed analysis of the cellulosic component of bacterial biofilms has revealed that bacteria can produce chemically modified cellulose variants, such as acetylated cellulose produced by *Pseudomonas fluorescens*.^9^ In a foundational study, Cegelski and co-workers applied solid state nuclear magnetic resonance spectroscopy to discover and isolate a new type of chemically modified cellulose, which they termed phosphoethanolamine cellulose **1**, from a UPEC strain (Figure 2A).^10^ Structurally, this polysaccharide is composed of 1,4-β-linked glucose residues with approximately half of the C-6 alcohols functionalized by phosphoethanolamine (pEtN). Interestingly, this polymer is produced by several microbial species, including *E. coli* and *Klebsiella pneumoniae*. While the mechanistic basis for pEtN modification of cellulose was recently disclosed, a limited number of studies have investigated the functional role of phosphoethanolamine cellulose.^11−13^

**Figure 2:**
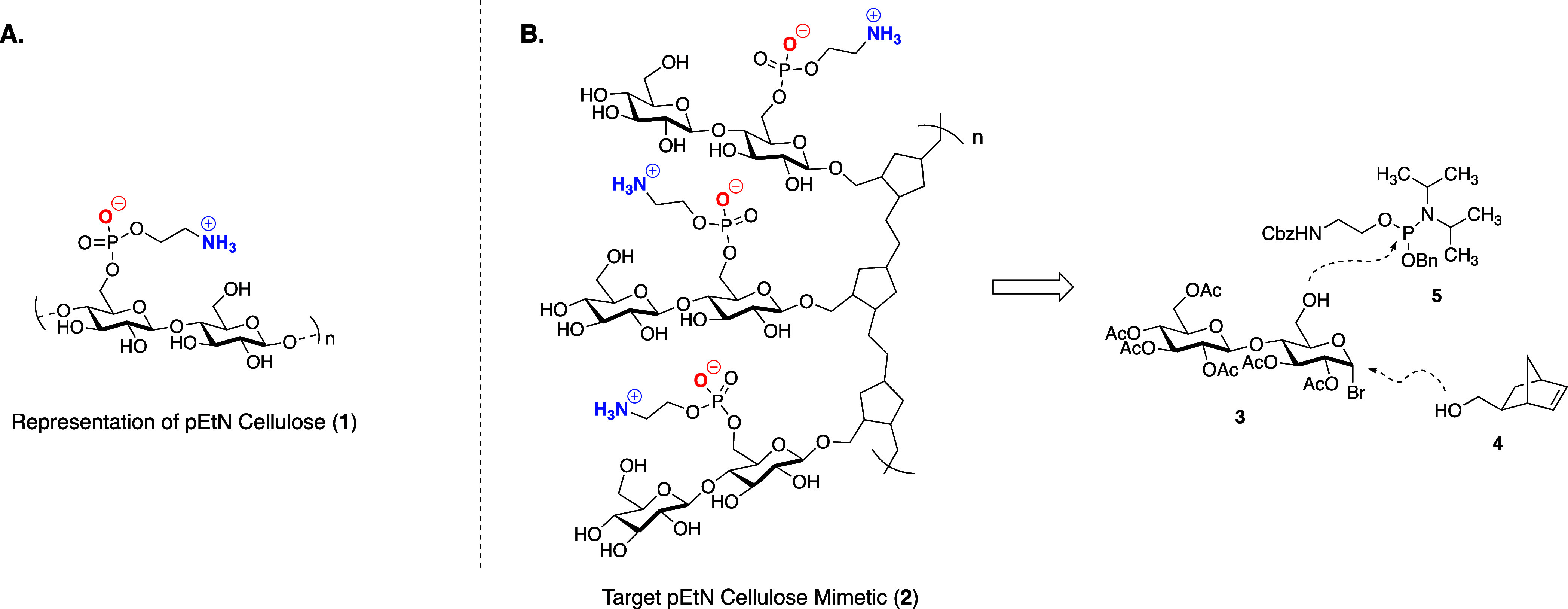
(A) Representative structure of pEtN cellulose. (B) Synthetic analysis of pEtN cellulose glycopolymers.

In a foundational study, Cegelski and co-workers discovered pEtN cellulose promotes adhesion of uropathogenic *E. coli* to epithelial tissue by acting as a “glue” between bacterial cells and curli fimbriae— extracellular amyloid fibers which facilitate bacterial attachment to tissue surfaces.^14,15^ Using colorimetric assays, our group later discovered that synthetic pEtN-cellobiose (the simplest repeating unit of pEtN-cellulose where *n* = 1) increased cellular adhesion of *E. coli* to an abiotic surface.^16^ Mechanistically, Congo red binding assays showed that culturing *E. coli* in the presence of pEtN cellobiose enhanced matrix production. Lastly, scanning electron microscopy visually confirmed the disaccharide increased the production of cell-associated fibers and biofilm architecture.

The discovery of a naturally occurring modified cellulose provides an opportunity to characterize how this modified glycan contributes to pathogenesis. Indeed, an excellent study by Delbianco and colleagues revealed that polysaccharide functionalization alters biofilm architecture and characteristics in an artificial model.^17^ Through application of a synthetic curli peptide-mimetic and various pEtN-modified glycans, the Delbianco team demonstrated that while short oligomers had little effect on fibrous structures, longer polysaccharides promoted growth of these structures. While exciting, a key gap remains in our ability to characterize how functionalized cellulose governs pathogenesis and virulence in a model of infection. Moreover, the correlation between mimetic fiber interactions with pEtN-cellulose and interactions in a whole cell model has yet to be evaluated. Furthermore, a major challenge in studying pEtN cellulose is the insolubility of the native polysaccharide and its isolation from cell culture. We hypothesized that the preparation of a synthetic, chemically defined pEtN cellulose mimetic would be a useful tool to further delineate the functional roles of this glycan. Moreover, these tool compounds could enable further exploration of bacterial cellulose materials and their influences on cellular interactions and biofilm formation (Figure 2B).^18,19^

## Results and Discussion

Since we previously observed that our synthetic pEtN cellobiose disaccharide enhanced cellular adhesion and biofilm formation to abiotic surfaces consistent with the native polysaccharide, we hypothesized that the tertiary structure of the native glycan was less critical to its biological function than its pEtN modification.^16^ Thus, at the planning stage, we envisioned the native polymer (simplified as structure **1**) could be adequately mimicked by structure **2** which contains an alkyl backbone modified by smaller pEtN functionalized cellobiose (Figure 2). To arrive at this structure, we proposed the synthesis of a pEtN cellobiose that incorporated a norbornene-based linker at the reducing end. The goal of this modification is to enable ring-opening metathesis polymerization (ROMP) which can be used to rapidly access glycopolymers with controlled molecular weights and narrow polydispersities.^20,21^ Our premise was based on the groups of Kiessling and Hsieh-Wilson who have both previously applied ROMP to access tool glycopolymers in a variety of settings.^22−26^ Glycopolymers prepared through ROMP are mimetics of the naturally occurring polymer, rather than direct duplicates, as the polymer scaffold will feature unnatural alkenes, aryl, or cyclopentyl functionality (depending on the nature of the starting monomer). Perhaps paradoxically, even those these functional groups are unnatural, their presence within the polymer backbone is advantageous as they increase structural rigidity which mitigates conformational entropy.

**Scheme 1:**
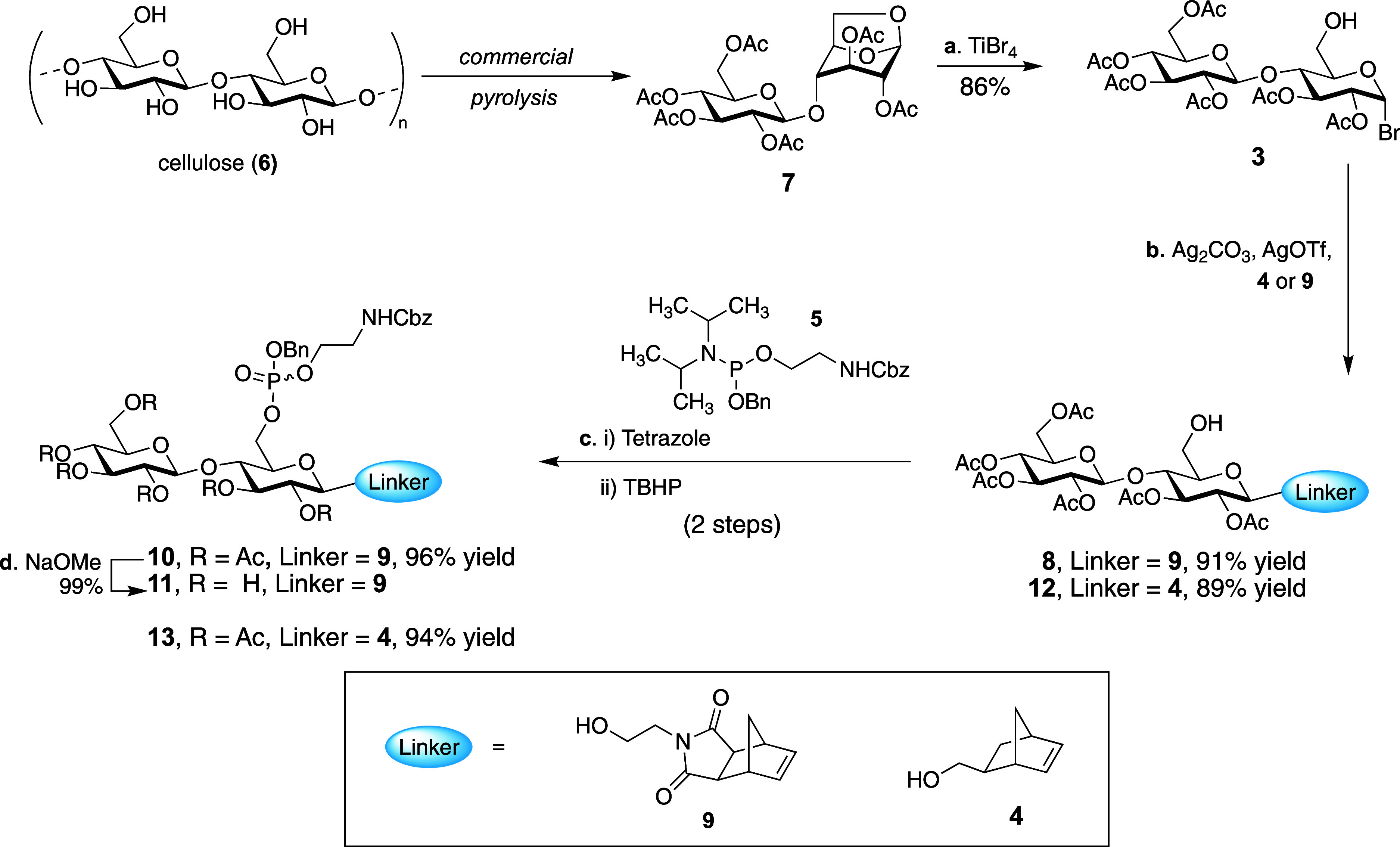
Synthesis of Functionalized Monomers for ROMP Reagents and conditions: (a) TiBr_4_ (2.5 equiv), CHCl_3_, 0 °C to reflux, 6 h, 86%; (b) Ag_2_CO_3_ (1.0 equiv), AgOTf (1.0 equiv), DCM, 3 Å MS, 0 to 23 °C, 91% for **8**, 89% for **12**; (c) (i) **5** (1.4 equiv), tetrazole (2.0 equiv), 3 Å MS, DCM, 0 °C, 1 h; (c) (ii) *t*-BuOOH (2.0 equiv), DCM, 0 °C, (96%, two steps for **10**, 94%, two steps for **13)**; (d) NaOMe (1.5 equiv), MeOH, 0 °C, 12 h, 99%.

The synthesis commenced from 1,6-anhydrocellobiose disaccharide **7** which is industrially produced from the pyrolysis of cellulose (Scheme 1). We hypothesized that the cyclic anomeric acetal would provide a convenient, inherent protecting group for the C-6 alcohol, negating the need for excessive protecting group manipulations that would lengthen the monomer synthesis. Cyclic anomeric acetals can be ring-opened through treatment with strong Lewis acids such as TiCl_4_ and lead to formation of the corresponding glycosyl chloride.^27^ While we found TiCl_4_ successfully formed the desired glycosyl chloride, this glycosyl donor performed poorly in glycosylation reactions. We attribute its unexpected stability to the disarming (electron withdrawing) effect of the acetate protecting groups. As a result, we opted to use TiBr_4_ for the acetal opening to produce glycosyl bromide **3**,  hich is a more reactive glycosyl donor.^28^ In the event, TiBr_4_-mediated acetal opening proceeded to afford the glycosyl bromide **3** in 86% yield. Subsequent glycosylation of this donor with *exo*-norbornenimide **9** under Koenigs–Knorr conditions proceeded smoothly to provide **8** in 90% yield.^29^ The next step in our synthesis was modification of the C-6 alcohol with the phosphoethanolamine arm. We selected the electrophilic phosphoramidite reagent **5** for this functionalization. Using tetrazole to activate the phosphoramidite, nucleophilic attack of the C-6 alcohol was facile. Subsequent oxidation of the P (III) center to the requisite P (V) center was achieved using *tert*-butyl hydroperoxide (TBHP) to afford the fully protected monomer **10**. Overall, the four-step synthetic sequence enabled rapid access to the desired ROMP precursor in 70% overall yield.

Next, we explored the ROMP of the functionalized monomer. Initial polymerization attempts were aimed at polymerizing deacetylated monomer **11** with the desire to minimize postpolymerization deprotection steps. Toward this end, we investigated the polymerization in a MeOH/CH_2_Cl_2_ cosolvent system to fully solubilize the starting monomer. We explored Grubbs second and third generation catalyst at a 5 mol % loading for the initial polymerization screen. During these reactions, we observed the growing polymer formed an insoluble film which led to incomplete monomer consumption. We hypothesize that the growing polymer chain becomes progressively less soluble, thereby removing the propagating species from the solution of solubilized monomer which halts polymerization. To address this issue, we took note of emulsion polymerization conditions reported by Yu and co-workers.^30^ Under reported conditions, unprotected monosaccharides are polymerized using HG-II catalyst in combination with a phase-transfer reagent TBAB (tetra-butyl ammonium bromide) in a DCE/bis–tris solvent system. Unfortunately, in our hands, these experiments suffered from incomplete polymerization and led to polymers with high polydispersity values and unpredictable molecular weights.

**Scheme 2:**
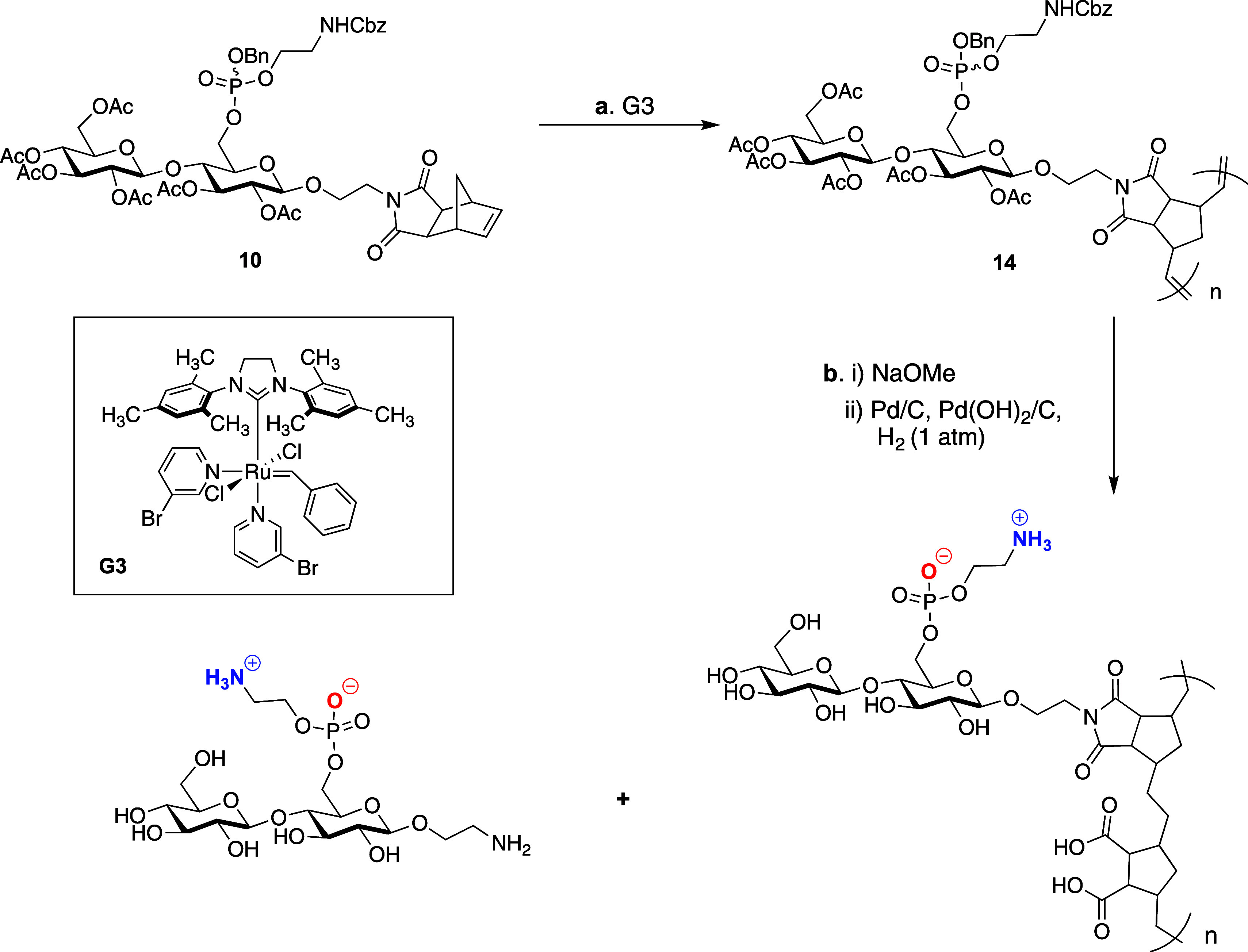
Polymerization and Deprotection of Norbornenimide-Derived pEtN Cellulose Glycopolymers Reagents and conditions: (a) Grubbs third generation catalyst, 1,2-DCE, 55 °C, 0.2 to 1 h, quant.; (b) (i) NaOMe (8.0 equiv), DCM/MeOH (1:2), 16 h; (b) (ii) Pd/C or Pd(OH)_2_/C (0.5 equiv), H_2_ (balloon), THF, MeOH, PBS (pH = 7.4), 23 °C, 4 d.

To circumvent these issues, we opted to polymerize the fully protected monomer **10** (Scheme 2). We selected the third Generation Grubbs catalyst for polymerization due to its rapid initiation kinetics.^31^ Under these conditions, polymerization proceeded rapidly and complete consumption of the monomer was observed. Next, we deprotected the polymers using a two-step sequence including deacetylation and global hydrogenolysis. Deacetylation proceeded smoothly under Zemplen conditions. However, when we investigated the hydrogenolysis using either Pd/C or Pearlman’s catalyst, we observed partial hydrolysis of the imide functionality resulting in cleavage of the disaccharide from the polymer backbone. We attribute the hydrolysis to the prolonged reaction time needed for complete hydrogenolysis (4 days) under aqueous conditions. Due to the unexpected lability of the imide functionality, we redesigned the polymer to incorporate a norbornene backbone derived from *exo*-5-norbornene-2-methanol **4** (Scheme 1). Fortunately, the short synthetic route we’d developed enabled us to rapidly prepare the desired functionalized monomer **13** (Scheme 1).

**Scheme 3:**
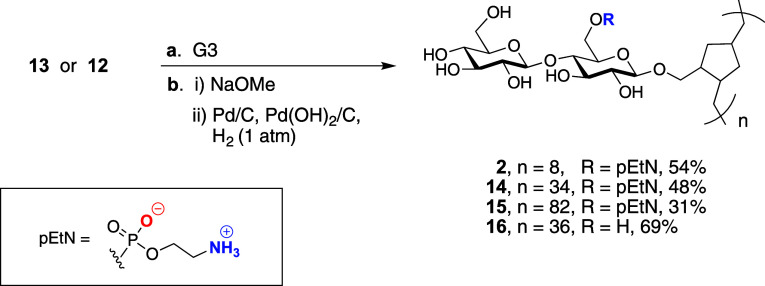
Polymerization and Deprotection of pEtN Cellulose Glycopolymers Reagents and conditions: (a) Grubbs third generation catalyst, 1,2-DCE, 55 °C, 0.2 to 1 h, quant.; (b) (i) NaOMe (8 equiv), MeOH, 16 h; (b) (ii) Pd/C (0.5 equiv), Pd(OH)_2_/C (0.5 equiv), H_2_ (balloon), THF, MeOH, PBS (pH = 7.4), 23 °C, 4 d, 31–69% (3 steps).

Excitingly, polymerization reactions with this monomer also proceeded rapidly, reaching completion within 1 h (Scheme 3). The resultant polymers were characterized by gel permeation chromatography (GPC) to determine degree of polymerization (DP) and polydispersity index (PDI) values. Polymerization of monomer **13** with Grubb’s catalyst at a loading of 10.0 mol % gave polymers with a DP = 8 and a PDI = 1.16. Lowering the catalyst loading gave polymers with longer chain lengths and slightly higher, but relatively narrow, polydispersities (see Table 1). Additionally, to investigate the importance of pEtN modification for this glycan, monomer **12** was polymerized to prepare a nonmodified cellulose glycopolymer (Scheme 3). Polymerization of monomer **12** at 2.5 mol % loading gave polymers with DP = 36 and PDI = 1.34 (see Table 1). Subsequently, the polymers were subjected to the two-step global deprotection sequence. Not surprisingly, the global deprotection of the large, zwitterionic glycopolymers proved to be the major challenge. After much experimentation, a 1:1 ratio of Pd/C and Pearlman’s catalyst (Pd(OH)_2_/C) was found to be the optimal hydrogenolysis catalyst system, requiring a full equivalent of catalyst for complete removal of all benzyl groups, Cbz groups, and reduction of backbone olefins.^27^ Furthermore, judicious selection of solvent was paramount to retain all intermediates in solution during the hydrogenolysis. A mixed solvent system of THF, MeOH, and phosphate-buffered solution pH = 7 (2:1:3) proved to be optimal. The pEtN cellulose glycopolymers **2** and **14–15** were obtained in 31–54% yield over three steps. Unsurprisingly, we observed that as the polymer chain length increased, our global deprotection yields decreased. This phenomenon is well-documented in carbohydrate oligomer synthesis.^32−34^ To the best of our knowledge, this represents the first reported synthesis of glycopolymers featuring a zwitterionic charge motif. **16** was obtained in 69% yield over 3 steps after global deprotection.

**Table 1:** Preparation of Protected Glycopolymers Using ROMP

entry	mol % catalyst	monomer	*n* (DP)	PDI
1	10.0	13	8	1.16
2	5.0	13	34	1.49
3	2.0	13	82	1.46
4	2.5	12	36	1.34

### pEtN Cellulose Glycopolymers Repress *E. coli* Biofilm Formation in a Size-Dependent Manner

**Figure 3:**
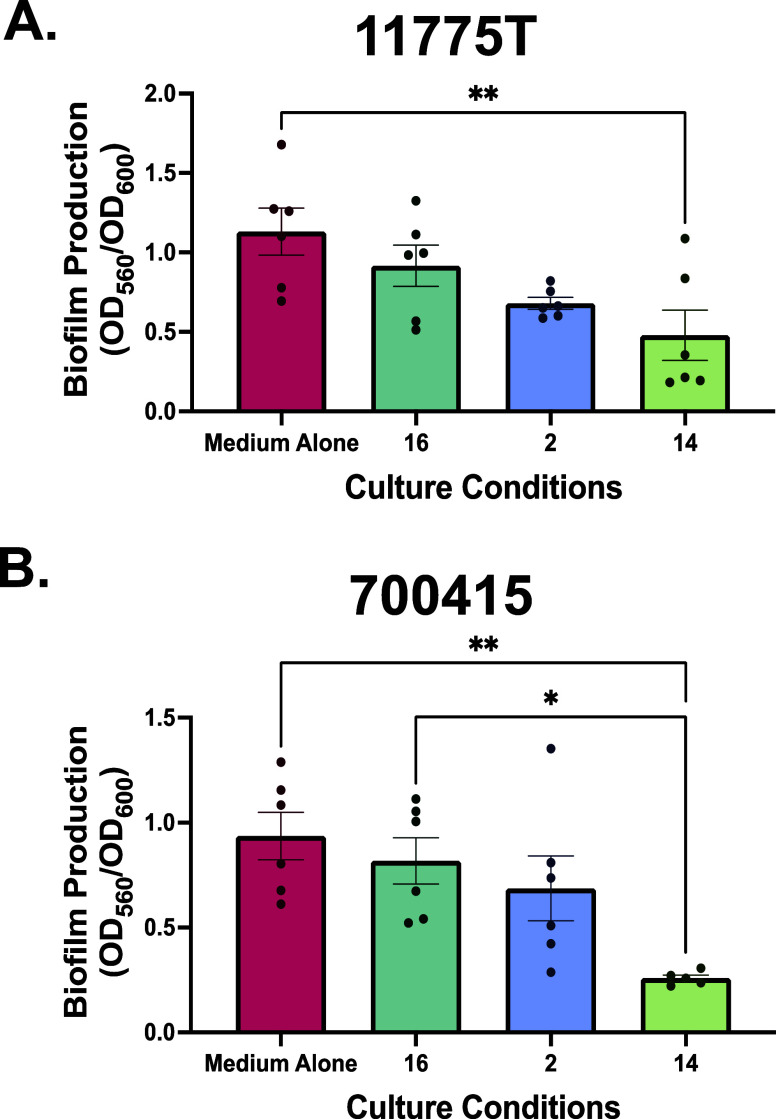
Effects of 2.5 mg/mL of pEtN glycopolymers on bacterial biofilm formation. Biofilm production was measured by crystal violet staining and spectrophotometric reading at OD_560_ normalized to total cell density as measured at OD_600_ at 24 h postinoculation. (A) Biofilm to biomass ratio (OD_560_/OD_600_) for *E. coli* 11775T in LB is shown. (B) Biofilm to biomass ratio (OD_560_/OD_600_) for *E. coli* 700415 in LB is shown. Data displayed represent the relative mZean biofilm/biomass ratio ± SEM of at least 3 independent experiments, each with 2 technical replicates. Significant inhibition of biofilm formation was determined by one-way ANOVA with *post hoc* Tukey’s test (****P < 0.00001).

Based on our earlier work with the pEtN cellobiose disaccharide, we hypothesized that the new glycopolymers would influence biofilm architecture of *E. coli*.^16^ We further hypothesized that different polymer lengths would induce phenotypic changes in virulence factor regulation associated with biofilm formation. In addition, we were interested in evaluating whether the phosphoethanolamine-modified glycopolymers would perform differently than a nonmodified cellulose control. To interrogate strain differences in bacterial behaviors with the modified cellulose polymers, we selected *E. coli* laboratory strain 11775T and UPEC strain 700415 for experiments. Biofilm production was expressed as the ratio of biofilm to biomass to account for any differences in cell density upon addition of polymers. When grown under normal conditions 11775T formed a robust biofilm. Treatment of 11775T with pEtN cellulose glycopolymer **14** (*n* = 34) resulted in an almost 3-fold reduction in biofilm production (*p* = 0.0085, one-way ANOVA with Tukey’s multiple comparison) (Figure 3A, and Supporting Information). The treatment with pEtN cellulose glycopolymer **2** (*n* = 8) also resulted in reduced biofilm formation, however these data were not statistically significant via one-way ANOVA. There was no significant change in biofilm production upon supplementation with nonmodified cellulose glycopolymer **16** (*n* = 36) compared to untreated controls.

Biofilm production was also analyzed for UPEC strain 700415 upon treatment with the synthetic glycopolymers. A similar trend was observed for 700415 where the nonmodified cellulose polymer **16** did not significantly influence biofilm production. However, treatment with pEtN cellulose glycopolymer **14** (*n* = 34) resulted in a marked 4-fold reduction in biofilm production compared with medium alone controls (*p* = 0.0038, one-way ANOVA, with Tukey’s multiple comparison) (Figure 3B). Treatment with glycopolymer **14** additionally resulted in significant decreases in biofilm forming abilities compared with the nonmodified cellulose glycopolymer **16** and pEtN glycopolymer **2** (*p* = 0.0175, one-way ANOVA with Tukey’s multiple comparison). While treatment with glycopolymer **2** (*n* = 8) did not produce a significant reduction in biofilm production, a trending reduction was observed between conditions featuring the two polymers compared to the no treatment control.

Interestingly, both strains exhibited iterative decreases in biofilm production with increasing pEtN glycopolymer length suggesting that, while virulence factor expression is differentially regulated between strains, pEtN glycopolymers uniformly influence biofilm formation. Notably, previous reports have demonstrated the efficacy of cationic, amine-containing polymers to disrupt biofilm formation.^35−37^ Generally, disruption of biofilm formation is thought to occur via electrostatic interactions with extracellular polymeric substances (EPS) and the outer membrane, which can lead to membrane rupture.^38^ However, our team previously demonstrated that pEtN cellobiose disaccharide enhanced biofilm formation.^16^ This suggested to us that the charged phosphoethanolamine moiety in our polymers was not simply disrupting EPS and membrane integrity. Taken together, these results suggest the biofilm modifying properties of synthetic pEtN cellulose glycopolymers are dependent upon polymer length.

### pEtN Cellulose Glycopolymers Decrease Amyloid Binding in *E. coli*

**Figure 4:**
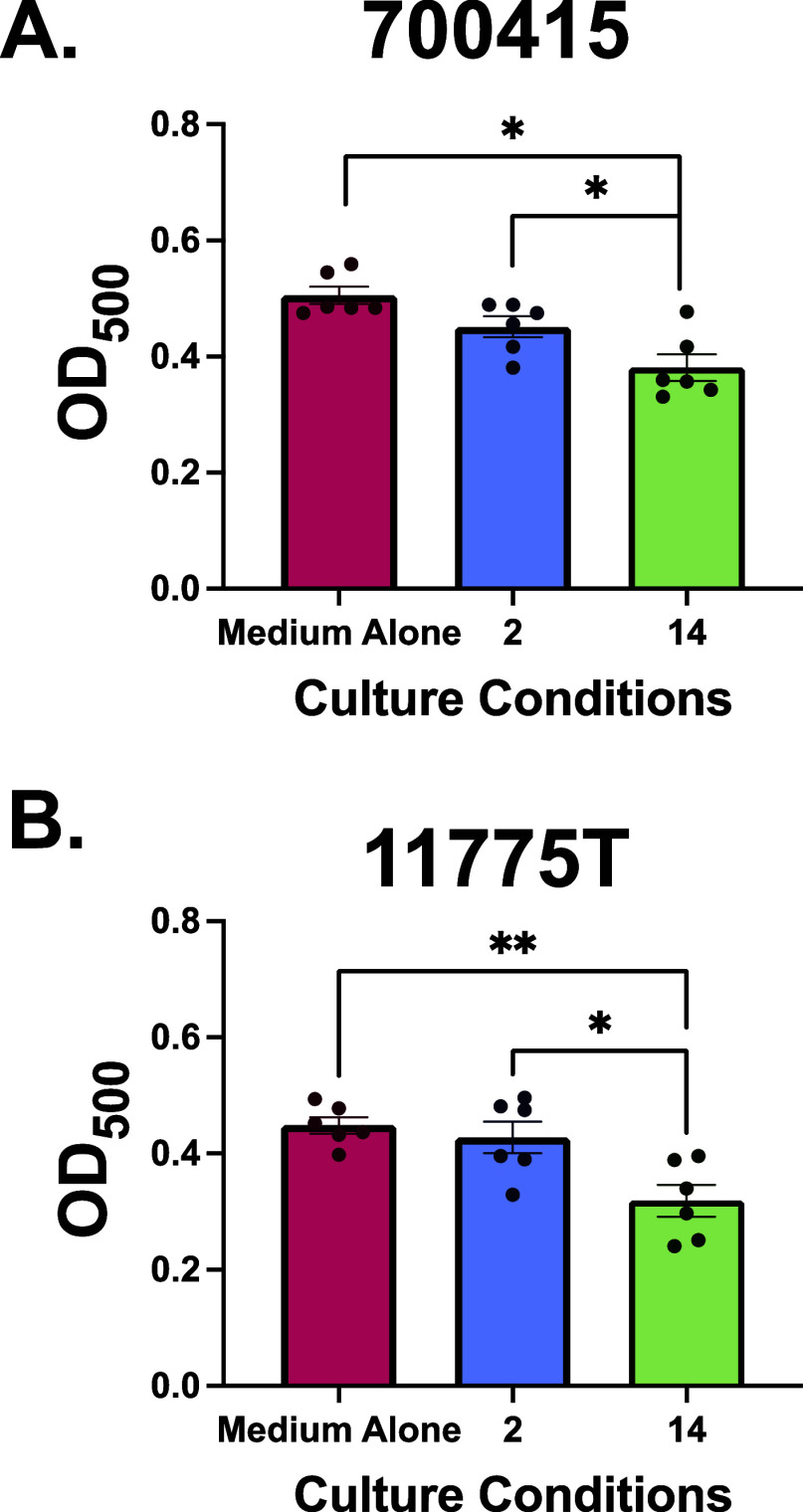
Effects of 2.5 mg/mL of pEtN glycopolymers on bacterial surface amyloid staining. Amyloid binding was measured by Congo red staining and spectrophotometric reading at OD_500_. (A) OD_500_. For *E. coli* 700415 in LB is shown. (B) OD_500._ For *E. coli* 11775T in LB is shown. Data displayed represent the relative mean ± SEM of at 2 independent experiments, each with 3 technical replicates. Significant differences in amyloid content were determined by one-way ANOVA with *post hoc* Tukey’s test (****P < 0.00001).

Consequently, we were interested in better understanding how the glycopolymers were inducing these changes. We postulated that the observed reduction in biofilm could be due to the glycopolymers binding and physically masking adhesion factors on the surface of the cell such as fimbriae, preventing surface attachment and biofilm formation. Our hypothesis was based on previous studies into pEtN cellulose that demonstrated the glycan acts as an adhesive agent between *E. coli* cells and curli fimbriae—amyloid fibers that comprise over 85% of the ECM in *E. coli* biofilms and are vitally important in facilitating attachment to a surface and cells within the biofilm.^14,39^ In addition, Delbianco and co-workers have shown that longer pEtN modified oligosaccharides coassemble more effectively to form extensive fibril networks with a synthetic peptide than shorter oligosaccharides.^17^ These studies suggest that pEtN cellulose has an affinity for fimbriae, which, in our case, could lead to inhibition of fimbriae-mediated attachment of cells to the surface via competitive binding. To determine whether our glycopolymers bind to these bacterial amyloids, we employed a Congo red binding assay. Congo red dye binds to amyloid fibers and cellulose, two major constituents of the ECM.^40,41^ Thus, we anticipated that pEtN glycopolymers could inhibit Congo red binding to these structures. Briefly, *E. coli* was grown in either medium alone or media supplemented with glycopolymer. After overnight incubation, cells were pelleted, washed to remove any remaining glycopolymer to eliminate any confounding binding to polymer, and treated with Congo red dye. Coculture of *E. coli* 700415 grown in media supplemented with glycopolymer **14** resulted in a significant decrease in Congo red binding when compared to untreated controls (*p* = 0.0008, one-way ANOVA with Tukey’s multiple comparison), while the shorter pEtN polymer **2** did not significantly reduce binding (Figure 4A). Similarly, coculture of *E. coli* 11775T grown in media supplemented with glycopolymer **2** did not exhibit a significant reduction in Congo red staining. However, treatment with glycopolymer **14** resulted in a significant reduction in Congo red staining (*p* = 0.0040, one-way ANOVA with Tukey’s multiple comparison) (Figure 4B). While these results are consistent with the hypothesis that our pEtN glycopolymers disrupt bacterial adhesion through binding and masking of bacterial fimbriae, a reduction in the prevalence of cell surface amyloid fibers could also result in decreased Congo red binding, biofilm formation and adherence in vitro. To distinguish between these possibilities, we employed high-resolution field emission scanning electron microscopy to visualize changes in cell-associated fibers.

## Single Electron Microscopy Analysis

**Figure 5:**
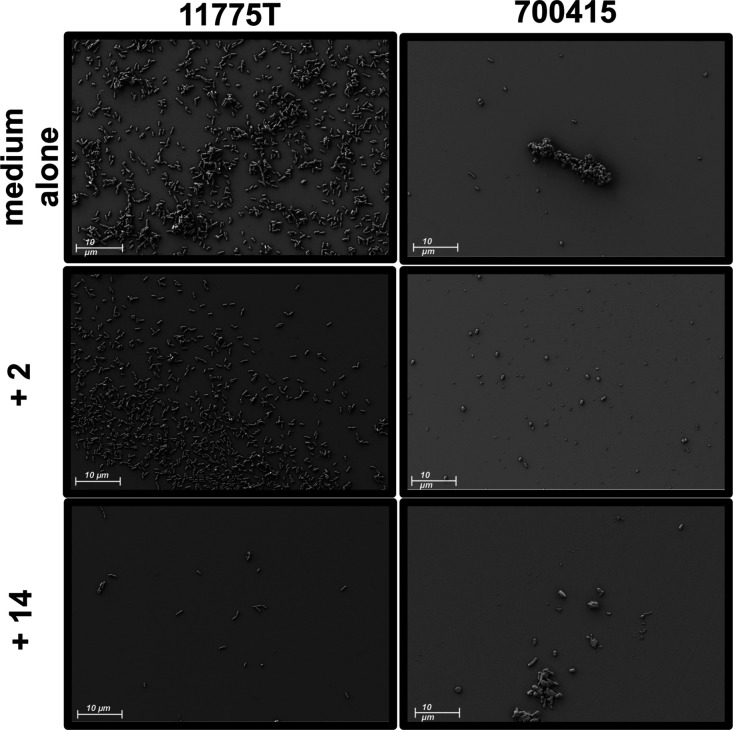
High resolution field-emission gun scanning electron microscopy analyses of *E. coli* biofilms. *E. coli* strains were cultured on glass coverslips in LB medium alone or supplemented with 2.5 mg/mL of each glycopolymer individually in static conditions to promote bacterial adherence andbiofilm formation. The addition of pEtN glycopolymers diminished bacterial biofilm formation.

**Figure 6:**
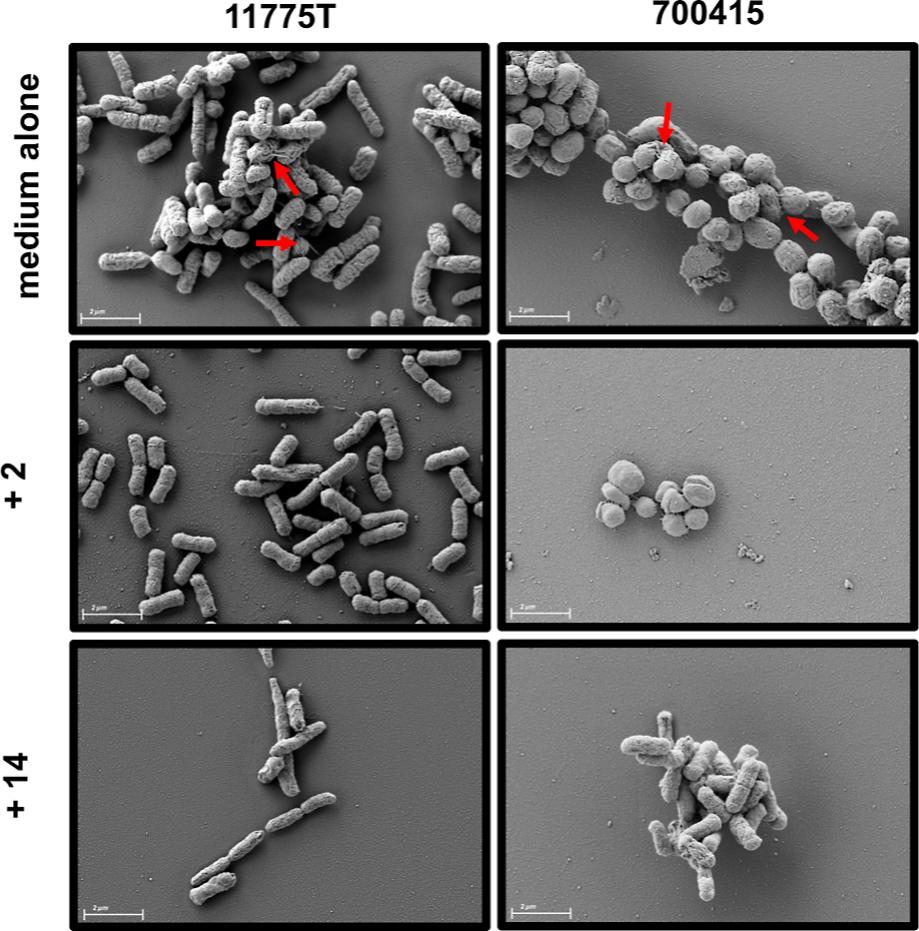
High resolution field-emission gun scanning electron microscopy analyses of *E. coli* cell surface appendages. *E. coli* strains were cultured on glass coverslips in LB medium alone or supplemented with 2.5 mg/mL of each glycopolymer individually in static conditions to promote bacterial adherence and biofilm formation. The addition of pEtN glycopolymers diminished bacterial surface appendage occurrence, indicated with red arrows.

*E. coli* strains were grown in LB media alone or supplemented with glycopolymer **2** or glycopolymer **14** in static conditions to promote biofilm formation. Electron microscopic analyses revealed decreases in sessile life and morphological changes in cell surface appendages upon treatment with the synthetic glycopolymers. The occurrence of coordinated cellular communities significantly diminished as cells were treated with increasingly larger pEtN glycopolymers (Figure 5). Between both strains cellular interactions decreased as polymer size increased across treatments. Further, upon closer examination, there is a distinct lack of cell surface appendages as pEtN glycopolymer size increases (Figure 6). Without treatment, cells from each strain are decorated with cell surface appendages of varying lengths. These results are consistent with the observed decrease in Congo red staining; however, these data instead support the notion that pEtN glycopolymers are inducing changes in surface adhesion factor prevalence, not simply masking surface adhesion factors. These structures are critical to surface adherence/attachment as well as cell-to-cell signaling, further corroborating the antiadhesive effects of these glycopolymers. Preliminarily, we hypothesize that these appendages could be type 1 and type 4 pili, both of which are implicated in adherence and biofilm structure.^1−3,38^

**Figure 7:**
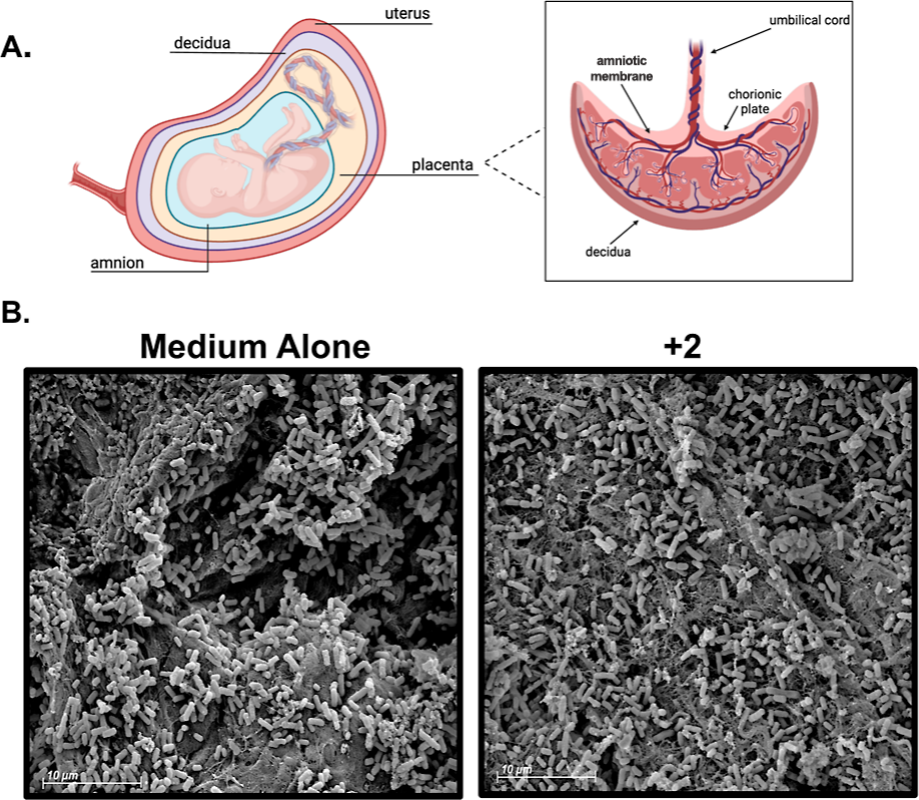
pEtN-modified cellulose glycopolymer reduces bacterial adherence to the choriodecidual face of the EPM in an ex vivo model. (A) Schematic of human gestational membranes. Human fetal tissues were collected from healthy term, nonlaboring Cesarian section deliveries, membranes removed, and treated with 2.5 mg/mL of 2 (*n* = 8 glycopolymer). (B) High resolution field emission gun scanning electron microscopy analyses of *E. coli* 700415 adherence on gestational membranes. *E. coli* 700415 was cultured on human gestational membranes alone or supplemented with 2.5 mg/mL of 2 (*n* = 8 glycopolymer). The addition of the pEtN-modified glycopolymer prevented adherence to and biofilm formation on ex vivo human gestational membranes.

It is well understood that adherence to abiotic surfaces is not always indicative of biotic surface adhesion, a vitally important step in colonizing a host.^43^ Because of this, we sought to understand how our glycopolymers performed in a more translational model of infection. Our lab has previously developed an ex vivo model of human extraplacental gestational membrane (EPM) tissue-bacterial infection.^44^ This model seemed fitting as UPEC strains are the main bacterial cause of UTIs, an infection presentation that is common during pregnancy and is often associated with negative outcomes for both the mother and developing fetus.^45^ It is well established that during these infections, pili are required for initial attachment of UPEC to bladder epithelial tissue via recognition of mannose residues on the tissue surface.^46,47^ Gestational membrane tissues similarly express mannose residues which may serve as sites of attachment for ascending UPEC in the genitourinary tract.^42,48^ Therefore, we hypothesized that pEtN glycopolymer treatment would similarly reduce bacterial adhesion to the membrane due to a lack of *E. coli* surface appendages. Briefly, UPEC strain 700415 was cultured on ex vivo human fetal tissues for 24 h with or without 2.5 mg/mL of glycopolymer **2**. The tissues were visualized via FEG-SEM to evaluate changes in biofilm morphology (Figure 7). Just as observed on the plastic surfaces, the pEtN glycopolymer qualitatively reduced *E. coli* bacterial adherence and colonization to the maternal choriodecidual face of the EPM (Figure 7).

We were intrigued by these findings as previous studies into the impacts of pEtN cellulose on biofilm architecture have linked pEtN cellulose to the production of matrix components and attachment of curli fimbriae.^14,16^ Indeed, we have previously shown that a pEtN cellobiose disaccharide promoted biofilm formation and appendage production. Based on this foundation, we hypothesized that the pEtN mimic glycopolymers would promote increased adhesion and subsequent biofilm formation. Contrary to our hypothesis, pEtN cellulose glycopolymers possess antibiofilm activity and decrease appendages and cell–cell communication. One potential explanation for these observed differences is that *E. coli* are able to utilize the previously reported pEtN cellobiose disaccharide as a carbon source, inducing bacterial growth and biofilm formation.^49,50^

## Conclusion

Overall, it is evident that our knowledge of the function of modified cellulosic polysaccharides and their mimics remain relatively under-explored, especially given the vast number of bacterial species that are known to produce them. Bacteria have evolved complex mechanisms to regulate expression of surface proteins and polysaccharides in response to external stimuli.^51^ Our results show that biofilm formation and adhesion of *E. coli* are reduced on abiotic and biotic surfaces in response to pEtN glycopolymers. SEM analyses revealed that pEtN glycopolymer treated cells are nonfimbriated. In future studies, we aim to further validate the mechanism by which our pEtN glycopolymers are modulating the occurrence of surface adhesins and biofilm formation through transcriptomics and proteomics, in hopes of unveiling novel uses for carbohydrate materials. Additionally, we aim to determine whether these observations are species specific by exploring the impact of our glycopolymers on other pEtN cellulose producers. Lastly, efforts are underway to expand the library of synthetic pEtN cellulose glycopolymers to better understand the influence of saccharide chain length and pEtN modification pattern. These results will be reported in due time.

## Methods

### Bacterial Strains and Culture Conditions

The bacterial strains used in this study were all *E. coli* strains: ATCC 11775T, a type strain urogenital isolate (serovar O1:K1:H7) and ATCC 700415, a urogenital isolate (serovar O4:H5), both of which harbor the genetic loci for curli production. *E. coli* strains were grown on tryptic soy agar plates supplemented with 5% sheep blood (blood agar plates) at 37 °C in ambient air overnight. The strains were subcultured from the blood agar plates into 5 mL of Luria–Bertani broth (LB) and incubated under shaking conditions at 180 rpm at 37 °C in ambient air overnight. Following overnight incubation, bacterial density was quantified through absorbance readings at an optical density at 600 nm (OD_600_) using a Promega GloMax-Multi Detection System plate reader. Bacterial numbers were determined using the predetermined coefficient of 1 OD_600_ = 109 cfu/mL.

### Bacterial Biofilm Assays

*E. coli* was grown overnight as described above and used to inoculate fresh LB at a multiplicity of infection (MOI) 106 colony forming units (cfus) per 100 μL of growth medium in 96 well tissue culture treated, sterile polystyrene plates. Both glycopolymers and unmodified cellulose were dissolved in DI water filtered through a 0.2 μm syringe filter. All compounds were added to achieve a final concentration of ca. 2.5 mg/mL, a therapeutic concentration that has been used in previously published experiments.^16^ Bacteria grown in LB in the absence of any treatments served as the control. Cultures were incubated under static conditions at 37 °C in ambient air for 24 h. Bacterial growth was quantified through absorbance readings at an optical density of 600 nm (OD_600_). Following growth quantification, the culture medium was removed, and wells were washed gently with phosphate buffered saline (PBS, pH 7.4) to remove nonadherent cells. The remaining biofilms were stained with a 10% crystal violet solution for 25 min. Following staining, wells were washed with PBS and allowed to dry at room temperature for at least 30 min. The remaining crystal violet stain was solubilized with 200 μL of 80% ethanol/20% acetone solution. Biofilm formation was then quantified through absorbance readings at an optical density of 560 nm (OD_560_). Results are expressed as biofilm/biomass ratios (OD_560_/OD_600_).

### Congo Red Assays

*E. coli* was grown overnight as described above and used to inoculate fresh LB at a MOI 106 cfus per 500 μL of growth medium in eppendorff tubes. Both glycopolymers were dissolved in DI water filtered through a 0.2 μm syringe filter and were added to achieve a final concentration of ca. 2.5 mg/mL. Bacteria grown in LB in the absence of any treatments served as the control. Cultures were incubated under static conditions at 37 °C in ambient air for 24 h. The following day samples were centrifuged at 500*g* for 5 min, supernatants removed, and cells washed 3 times with 1× PBS. Cells were then stained with ca. 0.1 mg/mL of Congo red in 1× PBS and incubated under shaking conditions at 37 °C in ambient air for 1 h. Cells were pelleted, supernatants removed, and pellet was washed with 1× PBS. Cells were resuspended in 1× PBS and amyloid binding quantified through absorbance readings at an optical density of 500 nm (OD_500_).

### Scanning Electron Microscopy Analysis

*E. coli* was grown on plastic coverslips in 500 μL LB media in the presence or absence of ca. 2.5 mg/mL of glycopolymers. Cultures were grown under static conditions at 37 °C in ambient air for 24 h. The following day, samples were fixed in a solution of 2.0% paraformaldehyde, 2.5% glutaraldehyde in a 0.05 M sodium cacodylate buffer at pH 7.4 for 24 h as previously described.^4^ After primary fixation, samples were subjected to sequential dehydration with increasing concentrations of ethanol and dried at the criticl point using a Tousimis Critical Point Dryer machine. Samples were mounted onto aluminum stubs, sputter coated with 20 nm of gold–palladium, and painted at the sample edge with a small stripe of colloidal silver to facilitate charge dissipation. Samples were imaged on a Zeiss Crossbeam 550 FIB-SEM at 2 keV using the in chamber secondary electron detector, images were acquired using Zeiss SmartSEM software.

### Gestational Membrane Coculture

Deidentified gestational membrane tissue samples were procured from term, nonlaboring Caesarean section-delivery live births at Vanderbilt University Medical Center with approval from the Vanderbilt University Medical Center Institutional Review Board (VUMC IRB #181998). 12 mm gestational membranes biopsy punches were isolated and cultured in RPMI 1640 medium (ThermoFisher, Waltham, MA) with 10% charcoal stripped fetal bovine serum (ThermoFisher) and 1% antibiotic/antimycotic solution (ThermoFisher) overnight at 37 °C in room air supplemented with 5% carbon dioxide. The membranes were washed 3 times, infected with 106 cfu/mL of *E. coli* in RMPI 1640 medium without antibiotics in the absence of treatment or supplemented with glycopolymer at a concentration of 2.5 mg/mL. A predetermined coefficient of bacterial density of 1 OD_600_ = 109 cfu/mL. Cocultured tissues were incubated at 37 °C in air supplemented with 5% carbon dioxide overnight and cells were fixed with 2.0% paraformaldehyde and 2.5% glutaraldehyde in 0.05 M sodium cacodylate buffer (Electron Microscopy Sciences, Hatfield, PA) for at least 12 h prior to processing for microscopy. Samples were viewed using an FEI Quanta 250 field-emission gun scanning electron microscope at 5 kEV with a spot size of 2.5.

### Statistical Analyses

Statistical analyses of biofilm quantifications were performed by Student’s *t*-test with Welch’s test and one-way ANOVA with Tukey’s post hoc test, comparing each strain’s growth in medium alone versus medium supplemented with HMOs. Analyses of bacterial growth in more than two conditions were performed using two-way ANOVA with Tukey’s post hoc test for multiple comparisons. All reported *P* values are adjusted to account for multiple comparisons. Quantitative culture results were analyzed with Student’s *t*-test. *P* values of ≤0.05 were considered significant. All data analyzed in this work were derived from at least three biological replicates. Statistical analyses were performed using GraphPad Prism 6 or 8 software (GraphPad Prism Software Inc., La Jolla, California) or Microsoft Excel.

## Supporting Information Available

Procedures for compound preparation; NMR and MS data for synthesized compounds; Figures S1–S4, GPC analyses of glycopolymers (PDF)
