# Supplementary material for: Synthesis of a Phosphoethanolamine Cellulose Mimetic and Evaluation of Its Unanticipated Biofilm Modulating Properties
Source: ACS Infect Dis. 2024 Aug 6;10(9):3245–55. doi: 10.1021/acsinfecdis.4c00267 (PMC11406534; doi:10.1021/acsinfecdis.4c00267)
Supplement: Supplementary file 1 — id4c00267_si_001.pdf [file id4c00267_si_001.pdf]

Supporting Information

Synthesis of a Phosphoethanolamine Cellulose Mimetic and Evaluation of its Unanticipated Biofilm Modulating Properties

C. Elizabeth Adams[1], Sabrina K. Spicer[1], Jennifer A. Gaddy[2,3,4] and Steven D. Townsend[1,4]*

[1]*Department of Chemistry, Vanderbilt University, Nashville, Tennessee 37235 (USA)*

[2]*Department of Medicine Vanderbilt University Medical Center, Nashville, Tennessee 37232 (USA)*

[3]*Department of Veterans Affairs, Tennessee Valley Healthcare Systems, Nashville, Tennessee 37212 (USA)*

[4]*Department of Pathology, Microbiology and Immunology, Vanderbilt University Medical Center, Nashville, Tennessee 37232 (USA)*

Email: steven.d.townsend@vanderbilt.edu

Table of Contents

I.	Title and authors	S1
II.	General procedure, materials, and instrumentation	S2
III.	Compound characterization and preparation	S3-11
IV.	^{1}H, ^{13}C, and ^{31}P NMR spectra of synthetic products	S12-S23
V.	GPC analysis of protected glycopolymers	S24-25
VI.	Quantification of bacterial biofilm (crystal violet- OD_{560})	S26
VII.	Supporting information references	S27

General Procedure: All non-aqueous reactions were performed in flame-dried or oven dried round-bottomed flasks under an atmosphere of nitrogen or argon, unless otherwise noted. Stainless steel syringes or cannula were used to transfer air- and moisture-sensitive liquids. Reaction temperatures were controlled using a thermocouple thermometer and analog hotplate stirrer. Reactions were conducted at room temperature (rt, approximately 23 °C) unless otherwise noted. The anhydrous solvents used in the reactions were obtained from an MBraun MB-SPS 800 anhydrous Solvent System. Solvent for chromatography were of analytical grade and distilled under reduced pressure prior to use. Commercially available reagents were obtained from Aldrich, Fisher, TCI, TRC, and Carbosynth. Flash column chromatography was conducted as described Still et. al. using silica gel 230-400 mesh or Silica RediSep Rf flash columns on a CombiFlash Rf automated flash chromatography system.1 Thin layer chromatography (TLC) was performed using glass backed 60-F254 silica gel plates obtained from Silicycle. Visualization of TLC plates was performed by UV (215 nm and 254 nm.

Instrumentation: ^{1}H NMR and ^{13}C NMR were recorded in the Vanderbilt Small Molecule NMR Facility on a Bruker 400 and 600 MHz. Structural assignments were made with additional information from gCOSY, gHSQC, and gHMBC experiments. Chemical shifts are reported in parts per million (ppm) of the δ scale. Spectra were recorded in CDCl3 by using the solvent residual peak chemical shift as the internal standard (CDCl3: S7 7.26 ppm 1 H, 77.0 ppm 13C) or in DMSO using the solvent as the internal standard in 1 H NMR (DMSO: 2.50 ppm 1 H) unless otherwise stated. 1 H NMR spectral data are presented as follows: Chemical shifts (δ ppm), multiplicity (s = singlet, d = doublet, dd = doublet of doublets, dq = doublet of quadruplet, ddd = doublet of doublet of doublet, t = triplet, q = quartet, p = pentet, br = broad, m = multiplet) coupling constants (Hz), integration. High-resolution mass spectra (HRMS) were obtained from the Department of Chemistry, Vanderbilt University using an LTQ-Orbitrap XL mass spectrometer.

(2*R*,3*R*,4*S*,5*R*,6*S*)-2-(acetoxymethyl)-6-(((2*R*,3*R*,4*S*,5*R*,6*R*)-4,5-diacetoxy-6-bromo-2-(hydroxymethyl)tetrahydro-2*H*-pyran-3-yl)oxy)tetrahydro-2*H*-pyran-3,4,5-triyl triacetate (3):

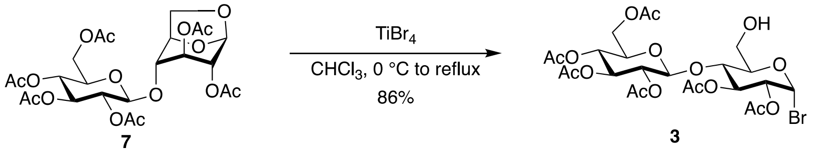

To a 100 mL round bottom flask was added commercially available **7** (2.00 g, 3.47 mmol, 1.00 eq.) and chloroform (30 mL). The mixture was cooled to 0 °C and $TiBr_4$ (3.19 g, 8.67 mmol, 2.50 eq.) was added. The mixture was stirred at 0 °C for 5 minutes, then a reflux condenser was attached and heated to reflux for 6 hours. Upon completion of the reaction, the mixture was cooled to room temperature and poured over a chilled (0 °C) saturated aqueous potassium sodium tartrate solution (100 ml). The mixture was extracted with EtOAc (3 x 50 mL), washed with H_2O (70 mL) and brine (70 mL). The organics were dried over anhydrous sodium sulfate, filtered, and concentrated in vacuo to afford glycosyl bromide **3** as a white solid (1.96 g, 2.98 mmol, 86% yield). ^{1}H NMR (600 MHz, CDCl3) δ 6.58 (d, *J* = 4.0 Hz, 1H), 5.52 (t, *J* = 9.4 Hz, 1H), 5.17 (t, *J* = 9.5 Hz, 1H), 5.08 (t, *J* = 9.7 Hz, 1H), 4.97 – 4.91 (m, 1H), 4.73 (dd, *J* = 10.0, 4.0 Hz, 1H), 4.68 (d, *J* = 8.1 Hz, 1H), 4.36 (dd, *J* = 12.5, 4.4 Hz, 1H), 4.08 – 3.95 (m, 3H), 3.91 (m, 2H), 3.73 – 3.66 (m, 1H), 2.09 (s, 3H), 2.09 (s, 3H), 2.08 (s, 3H), 2.04 (s, 6H), 2.01 (s, 3H), 1.98 (s, 3H). ^{13}C NMR (151 MHz, CDCl3) δ 170.59, 170.37, 170.13, 169.50, 169.35, 168.97, 100.41, 86.89, 75.35, 73.82, 73.03, 71.91, 71.70, 71.00, 69.57, 67.80, 61.66, 59.47, 20.72, 20.70, 20.66, 20.58, 20.57. HRMS (ESI) calcd. for $C_{24}H_{32}BrO_{16}$ (M - H)$^-$ 655.0874, found 655.0883.

(2*R*,3*R*,4*S*,5*R*,6*S*)-2-(acetoxymethyl)-6-(((2*R*,3*R*,4*S*,5*R*,6*R*)-4,5-diacetoxy-6-(((1*R*,2*S*,4*R*)-bicyclo[2.2.1]hept-5-en-2-yl)methoxy)-2-(hydroxymethyl)tetrahydro-2*H*-pyran-3-yl)oxy)tetrahydro-2*H*-pyran-3,4,5-triyl triacetate (12):

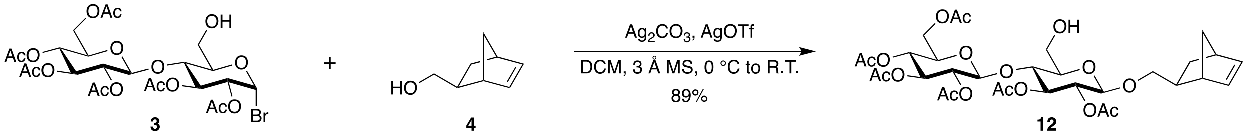

To a 25 mL round bottom flask was added bromide **3** (100 mg, 0.152 mmol, 1.00 eq.) and *rac*-exo-norbornene-2-methanol[1] **4** (75.6 mg, 0.608 mmol, 4.00 eq.). Dry dichloromethane (2.00 mL) and 3 Å molecular sieves were added and the mixture was stirred at R.T. for 1 hour. The mixture was then cooled to 0 °C and Ag_2CO_3 (42 mg, 0.15 mmol, 1.0 eq.) and AgOTf (39 mg, 0.15 mmol, 1.0 eq.) were added sequentially. The round bottom was foiled and allowed to warm to R.T and stirred 16 hours. Upon completion of the reaction, the mixture was filtered over celite, rinsing with DCM (40 mL). The organics were washed with sat. $NaHCO_3$. (15 mL), water (15 mL), brine (15 mL), and dried over anhydrous sodium sulfate and concentrated in vacuo. Flash chromatography of the crude material eluting with a gradient of Hexane/EtOAc (3:1 to 1:1) gave **12** as a mixture of diastereomers (95 mg, 0.14 mmol, 89% yield) as a white solid. ^1H NMR (600 MHz, CDCl3) δ 6.06 (s, 2H), 5.21 – 5.12 (m, 2H), 5.06 (t, J = 9.7 Hz, 1H), 4.96 – 4.85 (m, 2H), 4.63 (d, J = 8.1 Hz, 1H), 4.49 (t, J = 8.3 Hz, 1H), 4.36 (dd, J = 12.5, 4.3 Hz, 1H), 4.03 (d, J = 12.3 Hz, 1H), 3.96 – 3.88 (m, 2.5H), 3.79 – 3.72 (m, 1.5H), 3.71 – 3.66 (m, 1H), 3.52 (dd, J = 9.4, 6.6 Hz, 0.5H), 3.40 – 3.33 (m, 1.5H), 2.80 (s, 1H), 2.66 (d, J = 7.4 Hz, 1H), 2.08 (s, 3H), 2.05 – 2.00 (m, 12H), 1.98 (s, 3H), 1.70 – 1.60 (m, 2H), 1.34 – 1.29 (m, 1H), 1.29 – 1.24 (m, 2H), 1.24 – 1.17 (m, 1H), 1.07 (d, J = 11.8 Hz, 1H). ^{13}C NMR (151 MHz, CDCl3) δ 170.55, 170.27, 169.36, 168.99, 136.88, 136.82, 136.32, 101.07, 100.79, 100.57, 74.99, 74.89, 74.57, 73.00, 72.54, 71.85, 71.71, 67.84, 61.63, 60.25, 45.02, 44.81, 43.52, 43.34, 41.58, 38.71, 38.61, 29.72, 29.52, 29.32, 20.67, 20.62, 20.59, 20.57. HRMS (ESI) calcd. for $C_{32}H_{48}NO_{17}$ $(M + NH_4)^+$ 718.2922, found 718.2884.

(2*R*,3*R*,4*S*,5*R*,6*S*)-2-(acetoxymethyl)-6-(((2*R*,3*R*,4*S*,5*R*,6*R*)-4,5-diacetoxy-2-((((benzyloxy)(2-(((benzyloxy)carbonyl)amino)ethoxy)phosphoryl)oxy)methyl)-6-(((1*R*,2*S*,4*R*)-bicyclo[2.2.1]hept-5-en-2-yl)methoxy)tetrahydro-2*H*-pyran-3-yl)oxy)tetrahydro-2*H*-pyran-3,4,5-triyl triacetate (13):

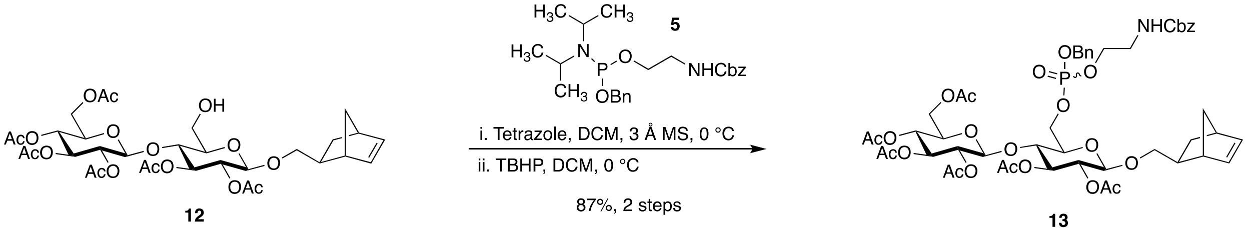

Molecular sieves (3 Å) were added to solution of **12** (1 eq., 600 mg, 0.56 mmol) and phosphoramidite **5**[2] (1.4 eq., 340 mg, 0.79 mmol) in dichloromethane (11 mL) and stirred for 1 h. A solution of tetrazole (2.0 eq., 79 mg, 1.1 mmol) in acetonitrile (5 mL) was added dropwise at room temperature. Upon completion of the reaction, a solution 5 M tert-butylhydroperoxide (2.0 eq, 0.2 mL, 1.1 mmol) was added at 0 °C. After 1 h, the solution was diluted with DCM and filtered over a pad of celite. The filtrate was washed with sat. $NaHCO_3$ (20 mL), water (20 mL), and brine (20 mL), dried over Na_2SO_4, and concentrated *in vacuo.* The crude residue was purified via flash column chromatography (1:1 hexanes/EtOAc to 1:2 hexanes/EtOAc) to give **13** as a mixture of diastereomers (690 mg, 87%, 0.487 mmol). ^{1}H NMR (600 MHz, $CDCl_3$) δ 7.44 – 7.29 (m, 10H), 6.03 (s, 2H), 5.49 (s, 1H), 5.26 – 5.07 (m, 6H), 5.06 – 4.99 (m, 1H), 4.93 – 4.83 (m, 2H), 4.60 (d, *J* = 8.0 Hz, 0.5H), 4.54 (dd, *J* = 8.0, 2.5 Hz, 0.5H), 4.47 (ddd, *J* = 10.6, 8.0, 2.5 Hz, 1H), 4.42 – 4.28 (m, 2H), 4.23 – 4.07 (m, 3H), 4.04 – 3.95 (m, 1H), 3.89 (td, *J* = 10.3, 6.1 Hz, 0.5H), 3.79 (q, *J* = 9.9 Hz, 1H), 3.75 – 3.64 (m, 1H), 3.56 – 3.39 (m, 4H), 3.33 (q, *J* = 9.8 Hz, 0.5H), 2.77 (s, 1H), 2.61 (d, *J* = 7.1 Hz, 1H), 2.13 – 1.90 (18H), 1.65 – 1.55 (m, 1H), 1.32 – 1.24 (m, 1H), 1.25 – 1.10 (m, 2H), 1.07 – 0.98 (m, 1H). ^{13}C NMR (151 MHz, $CDCl_3$) δ 170.53, 170.51, 170.24, 169.92, 169.90, 169.50, 169.35, 169.29, 169.03, 156.41, 136.86, 136.84, 136.78, 136.47, 136.31, 136.27, 136.25, 135.51, 135.47, 135.42, 128.88, 128.80, 128.77, 128.55, 128.53, 128.19, 128.17, 128.06, 128.01, 100.92, 100.61, 100.31, 100.29, 75.31, 75.18, 75.16, 74.79, 74.71, 74.46, 74.38, 73.27, 73.22, 72.32, 72.28, 71.80, 71.77, 71.70, 71.48, 71.44, 71.39, 69.92, 69.88, 69.70, 69.67, 67.73, 67.62, 67.12, 67.08, 67.04, 67.00, 66.79, 66.76, 65.14, 65.10, 65.04, 65.01, 61.51, 61.41, 44.95, 44.80, 44.78, 43.47, 43.44, 43.33, 43.30, 41.55, 41.36, 41.32, 38.63, 38.60, 38.51, 38.49, 29.41, 29.25, 20.66, 20.64, 20.57, 20.55, 20.53, 20.51. ^{31}P NMR (162 MHz, $CDCl_3$) δ 0.57, 0.48. HRMS (ESI) calcd. for $C_{49}H_{62}NNaO_{22}P$ $(M + Na)^+$ 1070.3399, found 1070.3359.

(2*R*,3*R*,4*S*,5*R*,6*S*)-2-(acetoxymethyl)-6-(((2*R*,3*R*,4*S*,5*R*,6*R*)-4,5-diacetoxy-6-(2-((3a*R*,4*R*,7*S*,7a*S*)-1,3-dioxo-1,3,3a,4,7,7a-hexahydro-2*H*-4,7-methanoisoindol-2-yl)ethoxy)-2-(hydroxymethyl)tetrahydro-2*H*-pyran-3-yl)oxy)tetrahydro-2*H*-pyran-3,4,5-triyl triacetate (8):

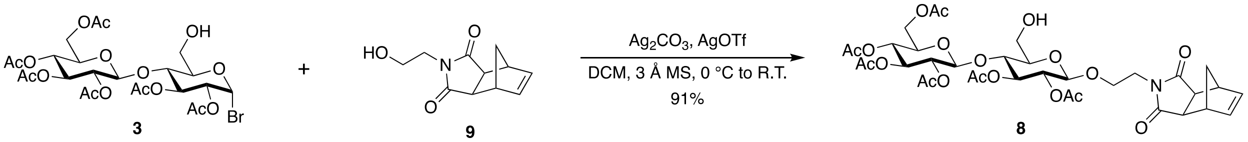

To a 15 mL round bottom flask was added bromide **3** (79 mg, 0.12 mmol, 1.00 eq.) and N-(hydroxyethyl)-cis-5-norbornene-exo-2,3-dicarboximide **9**[3] (250 mg, 1.2 mmol, 10.00 eq.). Dry dichloromethane (2.00 mL) and 3 Å molecular sieves were added and the mixture was stirred at R.T. for 1 hour. The mixture was then cooled to 0 °C and Ag_2CO_3 (33 mg, 0.12 mmol, 1.0 eq.) and AgOTf (31 mg, 0.12 mmol, 1.0 eq.) were added sequentially. The round bottom was foiled and allowed to warm to R.T and stirred 16 hours. The mixture was filtered over celite, rinsing with DCM (20 mL). The organics were washed with sat. $NaHCO_3$. (10 mL), water (10 mL), brine (10 mL), and dried over anhydrous sodium sulfate and concentrated in vacuo. Flash chromatography of the crude material eluting with a gradient of Hexane/EtOAc (3:1 to 1:1) gave **8** (86 mg, 0.11 mmol, 91% yield) as a white solid. ^{1}H NMR (600 MHz, $CDCl_3$) δ 6.22 (s, 2H), 5.09 (t, *J* = 9.5 Hz, 1H), 5.06 (t, *J* = 9.6 Hz, 1H), 5.00 (t, *J* = 9.8 Hz, 1H), 4.86 (dd, *J* = 9.6, 8.0 Hz, 1H), 4.72 (dd, *J* = 9.8, 7.9 Hz, 1H), 4.54 (d, *J* = 8.1 Hz, 1H), 4.45 (d, *J* = 7.9 Hz, 1H), 4.30 (dd, *J* = 12.5, 4.3 Hz, 1H), 3.96 (dd, *J* = 12.4, 2.3 Hz, 1H), 3.91 – 3.82 (m, 2H), 3.83 – 3.76 (m, 2H), 3.74 – 3.65 (m, 2H), 3.61 (ddd, *J* = 10.0, 4.3, 2.3 Hz, 1H), 3.50 (dt, *J* = 13.4, 4.9 Hz, 1H), 3.31 (dt, *J* = 9.8, 2.7 Hz, 1H), 3.20 (d, *J* = 1.8 Hz, 2H), 2.76 (dd, *J* = 9.6, 4.5 Hz, 1H), 2.61 (s, 2H), 2.01 (s, 3H), 1.97 (s, 3H), 1.96 (s, 3H), 1.94 (s, 3H), 1.93 (s, 3H), 1.91 (s, 3H), 1.46 – 1.41 (m, 1H), 1.27 (d, *J* = 9.8 Hz, 1H). ^{13}C NMR (151 MHz, $CDCl_3$) δ 178.16, 178.09, 170.53, 170.24, 169.85, 169.53, 169.34, 168.98, 137.87, 137.81, 101.07, 100.67, 75.01, 74.94, 72.97, 72.42, 71.82, 71.65, 71.29, 67.80, 65.42, 61.59, 60.13, 47.90, 47.81, 45.32, 45.30, 42.76, 39.07, 20.71, 20.66, 20.60, 20.56, 20.52. HRMS (ESI) calcd. for $C_{35}H_{45}NO_{19}$ $(M + H)^+$ 784.2664, found 784.2670.

(2*R*,3*R*,4*S*,5*R*,6*S*)-2-(acetoxymethyl)-6-(((2*R*,3*R*,4*S*,5*R*,6*R*)-4,5-diacetoxy-2-((((benzyloxy)(2-(((benzyloxy)carbonyl)amino)ethoxy)phosphoryl)oxy)methyl)-6-(2-((3a*R*,4*R*,7*S*,7a*S*)-1,3-dioxo-1,3,3a,4,7,7a-hexahydro-2*H*-4,7-methanoisoindol-2-yl)ethoxy)tetrahydro-2*H*-pyran-3-yl)oxy)tetrahydro-2*H*-pyran-3,4,5-triyl triacetate (10):

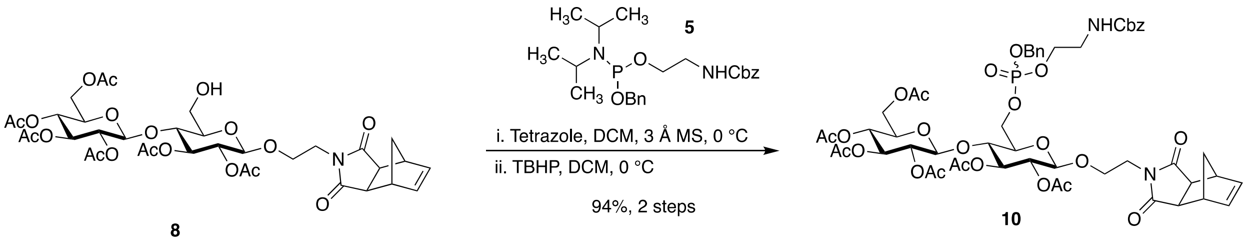

Molecular sieves (3 Å) were added to solution of **8** (1.0 eq., 159 mg, 0.20 mmol) and phosphoramidite **5**[2] (1.1 eq., 96 mg, 0.22 mmol) in dichloromethane (11 mL) and stirred for 1 h. A solution of tetrazole (1.2 eq., 79 mg, 0.24 mmol) in acetonitrile (0.72 mL) was added dropwise at room temperature. Upon completion of the reaction, a solution 5 M tert-butylhydroperoxide (5 eq, 0.18 mL, 1.0 mmol) was added at 0 °C. After 1 h, the solution was diluted with DCM and filtered over a pad of celite. The filtrate was washed with sat. $NaHCO_3$ (15 mL), water (15 mL), and brine (15 mL), dried over Na_2SO_4, and concentrated *in vacuo*. The crude residue was purified via flash column chromatography (1:1 hexanes/EtOAc to 1:2 hexanes/EtOAc) to give **10** as a white foam (215 mg, 94%, 0.19 mmol). ^{1}H NMR (600 MHz, $CDCl_3$) δ 7.43 – 7.28 (m, 10H), 6.23 (s, 2H), 5.58 (m, 1H), 5.14 – 5.06 (m, 6H), 5.03 (dt, *J* = 11.2, 9.7 Hz, 1H), 4.87 (ddd, *J* = 9.4, 8.0, 6.7 Hz, 1H), 4.80 (ddd, *J* = 9.5, 7.9, 1.5 Hz, 1H), 4.57 (d, *J* = 8.0 Hz, 0H), 4.50 (d, *J* = 8.1 Hz, 1H), 4.46 – 4.40 (m, 1H), 4.37 – 4.27 (m, 2H), 4.17 – 4.09 (m, 4H), 3.99 (d, *J* = 12.4 Hz, 0.5H), 3.96 (d, *J* = 12.3 Hz, 0.5H), 3.94 – 3.86 (m, 1H), 3.74 (td, *J* = 9.5, 2.8 Hz, 1H), 3.69 – 3.55 (m, 4H), 3.47 (d, *J* = 5.7 Hz, 4H), 3.21 (d, *J* = 13.8 Hz, 2H), 2.61 (s, 2H), 2.04 – 1.96 (18H), 1.93 (s, 1H), 1.45 (d, *J* = 9.9 Hz, 1H), 1.22 (d, *J* = 5.1 Hz, 1H). ^{13}C NMR (151 MHz, CDCl3) δ 177.87, 177.77, 177.74, 170.50, 170.48, 170.21, 169.78, 169.76, 169.45, 169.33, 169.27, 169.01, 168.99, 156.42, 137.82, 137.73, 136.52, 135.53, 128.88, 128.86, 128.80, 128.77, 128.53, 128.20, 128.17, 128.15, 128.10, 128.04, 100.29, 100.23, 75.19, 75.05, 73.32, 73.27, 72.90, 72.23, 72.21, 71.81, 71.77, 71.64, 71.10, 69.86, 69.82, 69.72, 69.68, 67.70, 67.61, 67.08, 67.03, 66.74, 64.98, 64.93, 64.84, 61.48, 61.39, 47.79, 47.76, 45.22, 45.18, 42.77, 41.33, 37.98, 37.94, 20.67, 20.63, 20.56, 20.54, 20.47. ^{31}P NMR (162 MHz, CDCl3) δ 0.66, 0.49. HRMS (ESI) calcd. for $C_{52}H_{64}N_2O_{24}P$ $(M + H)^+$ 1131.3587, found 1131.3579.

benzyl (2-(((benzyloxy)(((2R,3S,4R,5R,6R)-6-(2-((3aR,4R,7S,7aS)-1,3-dioxo-1,3,3a,4,7,7a-hexahydro-2H-4,7-methanoisoindol-2-yl)ethoxy)-4,5-dihydroxy-3-(((2S,3R,4S,5S,6R)-3,4,5-trihydroxy-6-(hydroxymethyl)tetrahydro-2H-pyran-2-yl)oxy)tetrahydro-2H-pyran-2-yl)methoxy)phosphoryl)oxy)ethyl)carbamate (11):

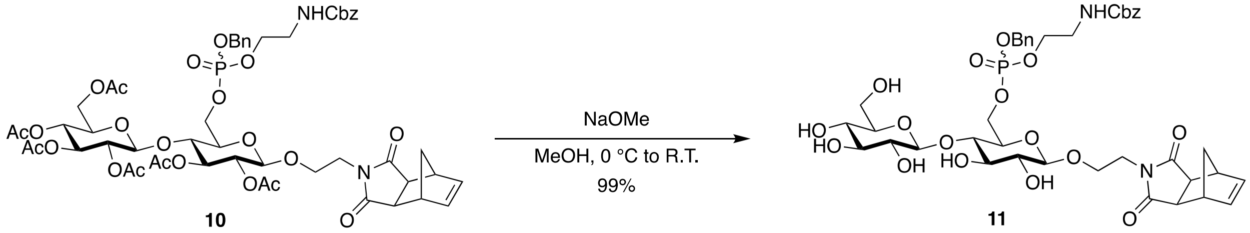

To a solution of **10** (1.0 eq, 0.36 mmol, 412 mg) in MeOH cooled to 0°C was added 1M NaOMe (1.5 eq, 0.55 mmol, 0.55 mL). The solution was stirred at room temperature for 10 h, then the reaction was quenched with Dowex 50XW8 resin until the pH was neutral. The solution was filtered over Celite and concentrated *in vacuo* to yield a mixture of diastereomers **11** as a white foam (310 mg, 0.35 mmol, 97%). ^{1}H NMR (600 MHz, MeOD) δ 7.48 – 7.27 (m, 10H), 6.29 (s, 2H), 5.18 – 5.07 (m, 5H), 4.50 – 4.44 (m, 1H), 4.35 (dd, J = 10.4, 7.7 Hz, 2H), 4.30 (d, J = 7.8 Hz, 1H), 4.12 (qt, J = 15.3, 7.5 Hz, 2H), 4.01 – 3.94 (m, 1H), 3.89 (ddd, J = 11.9, 4.9, 1.7 Hz, 1H), 3.79 – 3.62 (m, 4H), 3.62 – 3.57 (m, 1H), 3.52 (t, J = 8.9 Hz, 1H), 3.49 – 3.36 (m, 5H), 3.34 – 3.28 (m, 2H), 3.24 (ddd, J = 9.1, 7.7, 3.6 Hz, 1H), 3.21 (t, J = 8.4 Hz, 1H), 3.16 (dd, J = 5.9, 4.3 Hz, 2H), 2.69 (p, J = 6.9 Hz, 2H), 1.44 (dd, J = 10.1, 1.7 Hz, 1H), 1.36 (dd, J = 9.9, 2.1 Hz, 1H). 13C NMR (151 MHz, MeOD) δ 178.89, 178.72, 157.36, 137.56, 137.50, 136.88, 135.85, 135.81, 128.38, 128.36, 128.16, 127.84, 127.78, 127.56, 103.29, 102.53, 102.49, 79.01, 78.99, 76.77, 76.50, 74.60, 73.53, 73.10, 73.05, 69.94, 69.57, 69.51, 69.47, 66.66, 66.32, 66.19, 65.34, 61.05, 45.03, 45.00, 42.34, 40.73, 40.68, 38.00. HRMS (ESI) calcd. for $C_{40}H_{52}N_2O_{18}P$ $(M + H)^+$ 879.2953, found 879.2974.

General procedure for polymerization.

In a typical polymerization experiment, a 10 mL flame-dried round bottom was charged with monomer **12** or **13** (50.0 mg) and a small stir bar under the flow of argon. The monomer was dissolved in Ar-sparged $(CH_2Cl_2)_2$ and the desired amount of bis-pyridine stock solution in $(CH_2Cl_2)_2$ was added via syringe at room temperature. The reaction was heated to 55 °C and stirred at this temperature until TLC of the crude reaction mixture showed complete consumption of the starting monomer. The reaction was quenched with ethyl vinyl ether (0.3 mL) and stirred for 1 hour. The solvent was removed *in vacuo*.

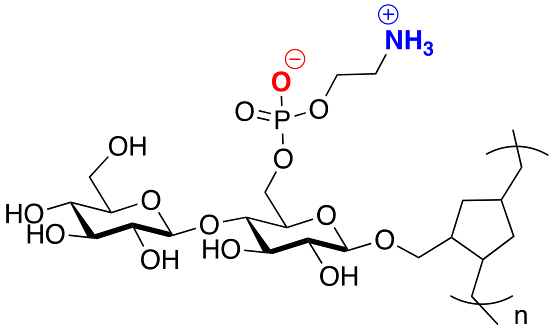

pEtN cellulose glycopolymers (2, 14, and 15).

The general polymerization procedure was used as described with monomer **13** (0.045 mmol). The ^{1}H NMR of the crude product showed the disappearance of the norbornene olefin protons at 6.0 ppm, further confirming the completion of the polymerization reaction. The resultant glycopolymers were characterized by gel permeation chromatography (GPC) to determine degree of polymerization (DP) and polydispersity (PDI). GPC was carried out in THF on two TSKgel GMHHR-M mixed bed columns (Tosoh Biosciences) connected in series with a refractive index detector, light scattering detector and viscometer (Viscotek). The dn/dc values were obtained for each injection assuming 100% mass elution from the column using Poly(styrene) (90K and 135K) as a calibration standard to confirm complete mass recovery. The DP and PDI values for polymerizations with different catalyst loadings are shown in **Table 1** and GPC traces are shown in **Figures S1-3**. The crude polymer was then dissolved in 1:1 DCM/MeOH (5 mL) then treated with excess NaOMe (0.5 M) and stirred for 16 h. The reaction was neutralized with formic acid until pH reached 7, then solvent evaporated. The crude mixture was dissolved in 2:1:3 (THF/MeOH/PBS Buffer) and added 10% $Pd(OH)_2/C$ (0.5 eq) and 5%

Pd/C (0.5 eq). The mixture was sparged with Ar, then H_2, and stirred under H_2 (balloon) for 4 d. The reaction mixture was filtered over celite and concentrated. ^{1}H NMR spectroscopy of the crude mixture was used to determine reaction completion as indicated by the disappearance of norbornene olefinic protons around 5 ppm and aromatic protons around 7.2 ppm. The crude residue was dissolved in de-ionized water and purified by size exclusion chromatography on G-25 Sephadex gel (100% H_2O) and lyophilized to afford pEtN cellulose glycopolymers **2** (54% yield), **14** (48% yield), or **15** (31% yield) as fluffy white solids. The yields are reported over three steps – polymerization, saponification, and hydrogenolysis. The purified, de-protected glycopolymers were analyzed by ^{1}H and ^{31}P NMR spectroscopy.

pEtN cellulose glycopolymers 2, 14, and 15: ^{1}H NMR (600 MHz, D2O) δ 4.60 (s, 1H), 4.46 (br, 1H), 4.32 – 4.06 (m, 4H), 3.93 (d, *J* = 11.8 Hz, 1H), 3.82 – 3.58 (m, 5H), 3.53 (t, *J* = 9.1 Hz, 2H), 3.42 (t, *J* = 9.1 Hz, 1H), 3.36– 3.27 (m, 5H), 1.86 (br, 4H), 1.37 (br, 6H), 0.75 (br, 1H). ^{31}P NMR (162 MHz, CDCl3) δ 1.29.

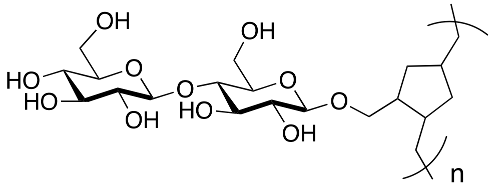

Cellulose glycopolymer (16).

The general polymerization procedure was used as described with monomer **12** (0.071 mmol). The resultant glycopolymer was characterized by gel permeation chromatography (GPC) to determine degree of polymerization (DP) and polydispersity (PDI). GPC was carried out in THF on two TSKgel GMHHR-M mixed bed columns (Tosoh Biosciences) connected in series with a refractive index detector, light scattering detector and viscometer (Viscotek). The dn/dc values were obtained for each injection assuming 100% mass elution from the column using Poly(styrene) (90K and 135K) as a calibration standard to confirm complete mass recovery. The DP and PDI values are shown in **Table 1** and the GPC trace is shown in **Figure S4**. The crude polymer was then dissolved in 1:1 DCM/MeOH (5 mL) then treated with excess NaOMe (0.5 M) and stirred for 16 h. The reaction was neutralized with formic acid until pH reached 7, then solvent evaporated. The crude mixture was dissolved in 2:1:3 (THF/MeOH/PBS Buffer) and added 10% $Pd(OH)_2/C$ (0.5 eq) and 5% Pd/C (0.5 eq). The mixture was sparged with Ar, then

H_2, and stirred under H_2 (balloon) for 4 d. The reaction mixture was filtered over celite and concentrated. ^{1}H NMR spectroscopy of the crude mixture was used to determine reaction completion as indicated by the disappearance of norbornene olefinic protons around 5 ppm and aromatic protons around 7-7.5 ppm. The crude residue was dissolved in de-ionized water and purified by size exclusion chromatography on G-25 Sephadex gel (100% H_2O) and lyophilized to afford **16** (69% yield) as a fluffy white solid. The yield for **16** is reported over three steps – polymerization, saponification, and hydrogenolysis. The purified, de-protected glycopolymer was analyzed by ^{1}H spectroscopy.

^{1}H NMR (600 MHz, D2O) δ 4.53 (s, 1H), 4.42 (br, 1H), 3.93 (d, *J* = 12.3 Hz, 2H), 3.87 (br, 1H) 3.78 – 3.72 (m, 2H), 3.69 – 3.60 (br, 2H), 3.56 – 3.48 (m, 3H), 3.43 (m, 2H), 3.35 (br, 2H), 1.95 – 1.73 (br, 4H), 1.46 – 1.22 (br, 7H).

^{1}H NMR of (600 MHz, CDCl3) of Compound 3

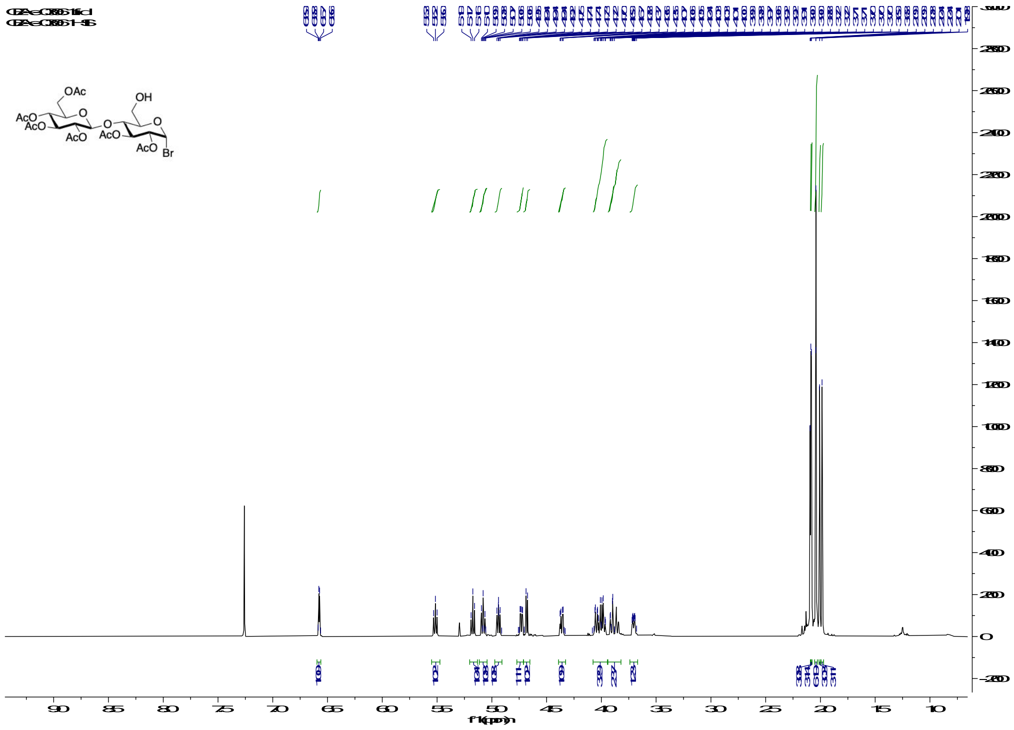

^{13}C NMR of (151 MHz, CDCl3) of Compound 3

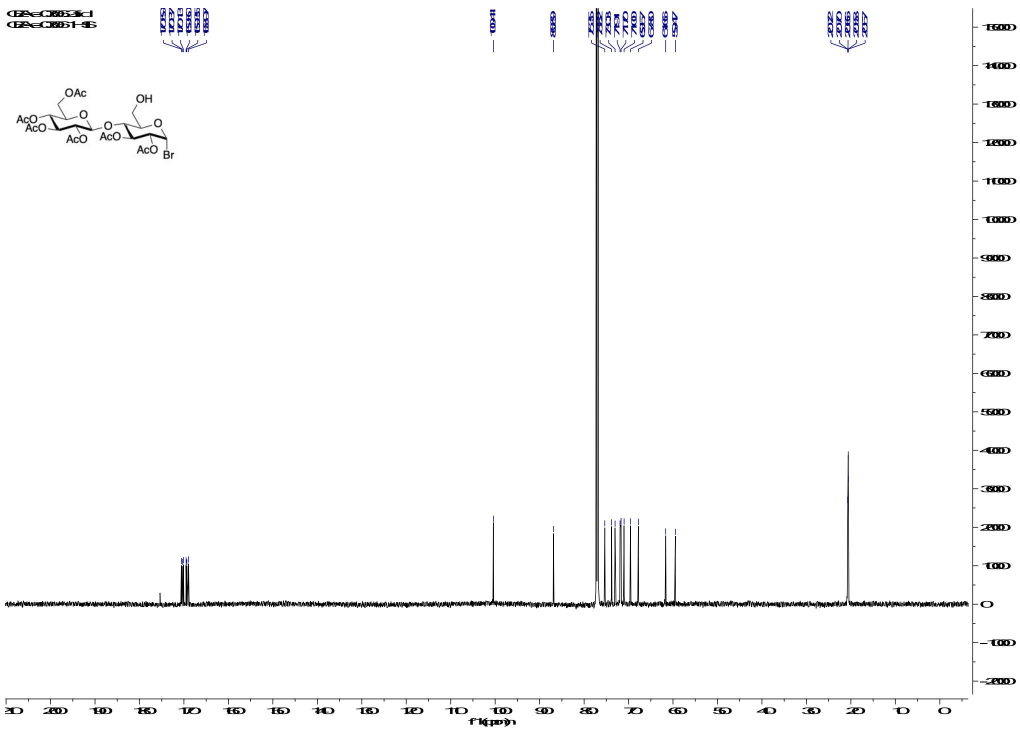

^{1}H NMR of (600 MHz, CDCl3) of Compound 12

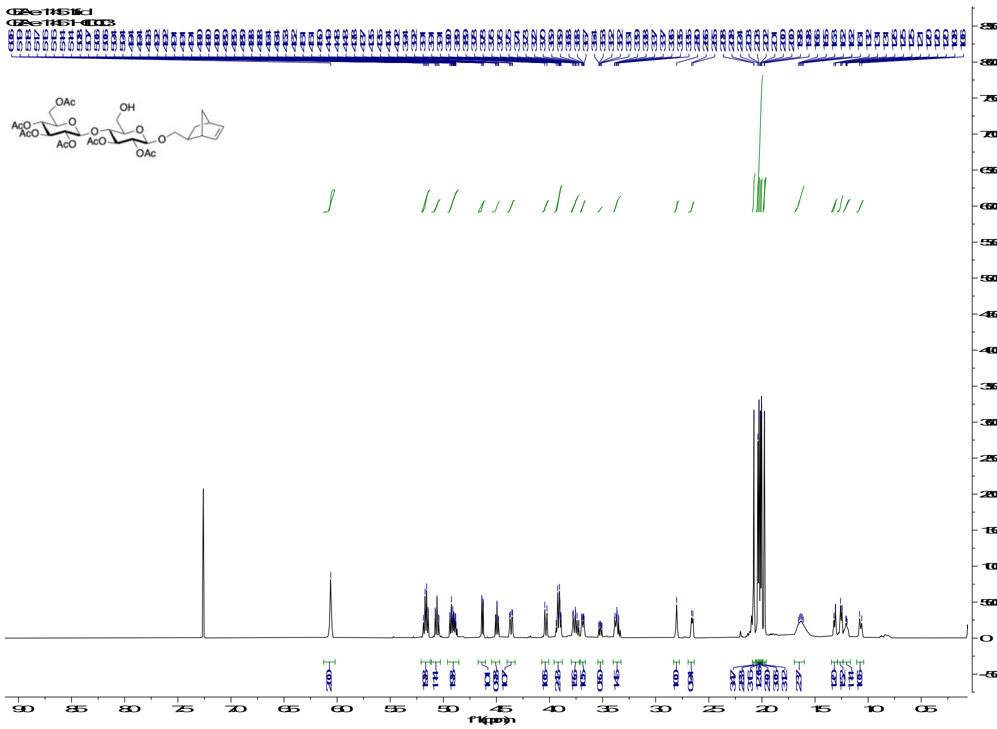

^{13}C NMR of (151 MHz, CDCl3) of Compound 12

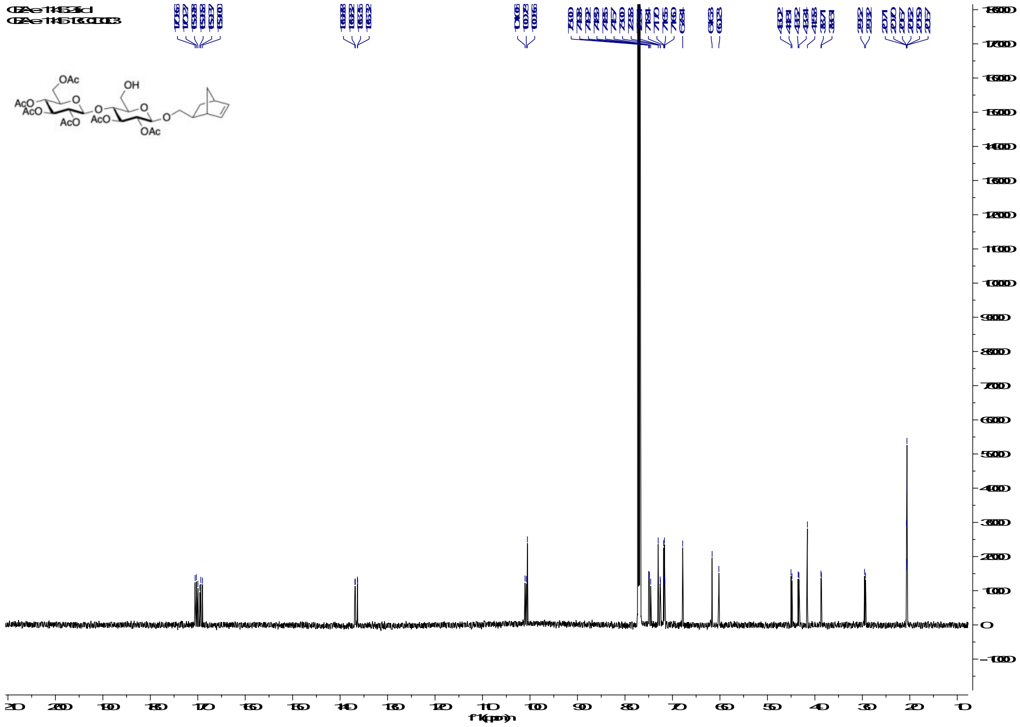

^{1}H NMR of (600 MHz, CDCl3) of Compound 13

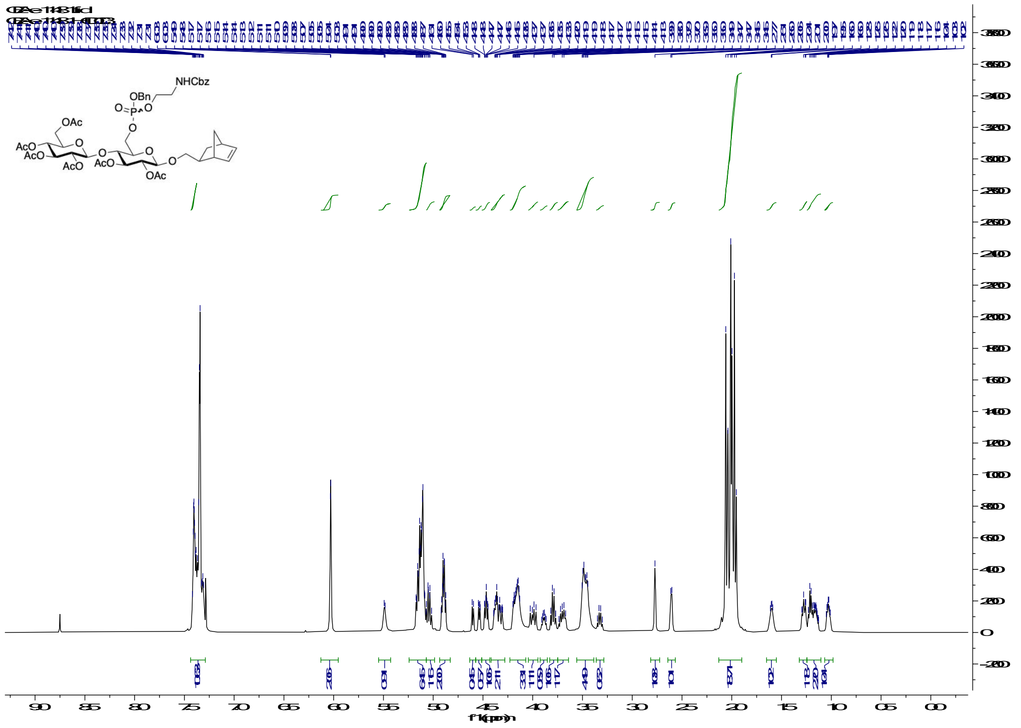

^{13}C NMR of (151 MHz, CDCl3) of Compound 13

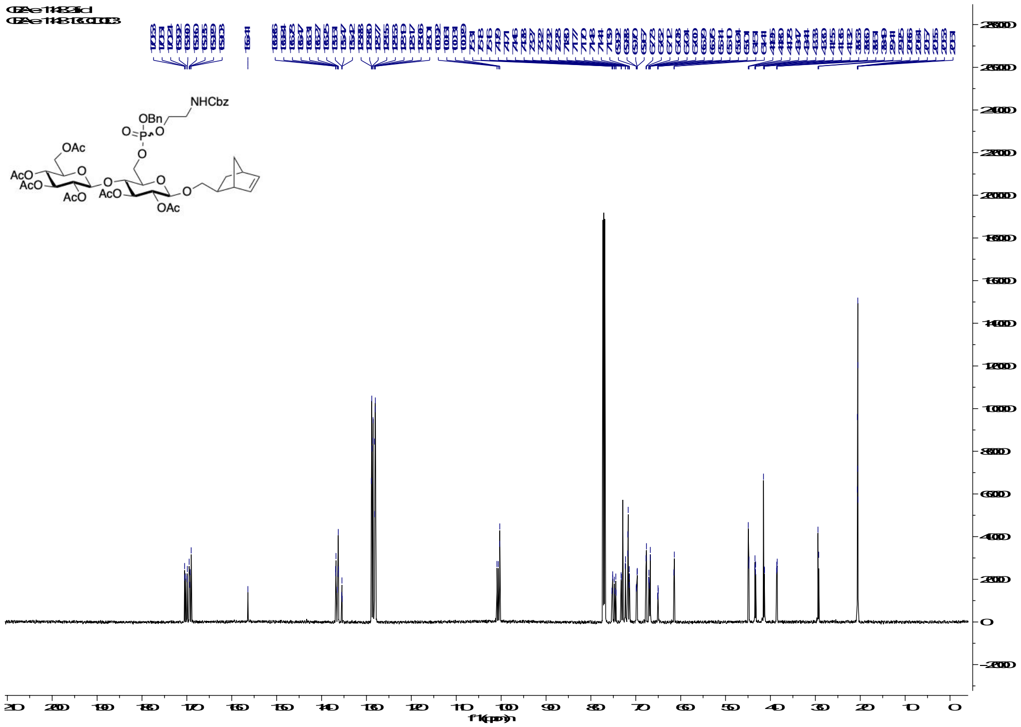

^{31}P NMR of (162 MHz, CDCl3) of Compound 13

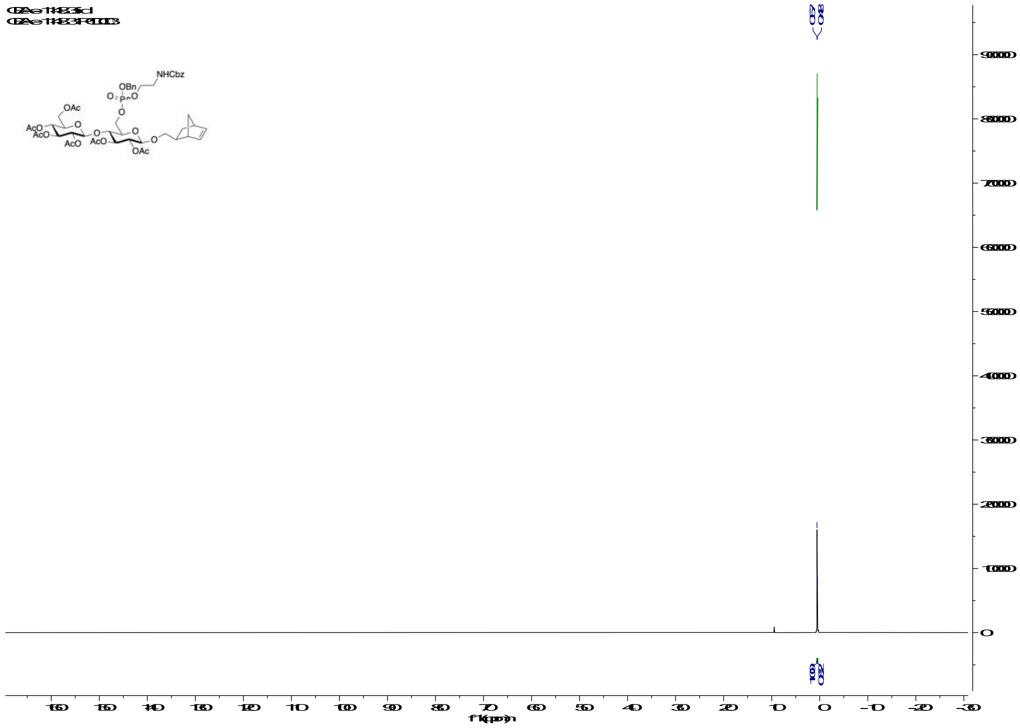

^{1}H NMR of (600 MHz, CDCl3) of Compound 8

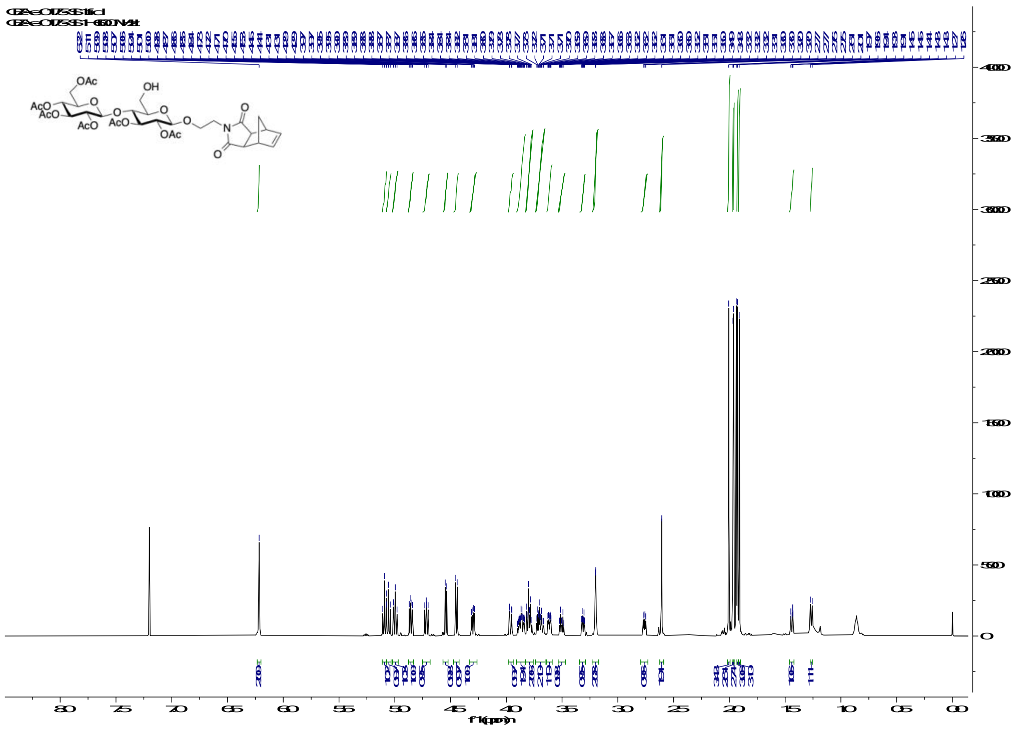

^{13}C NMR of (151 MHz, CDCl3) of Compound 8

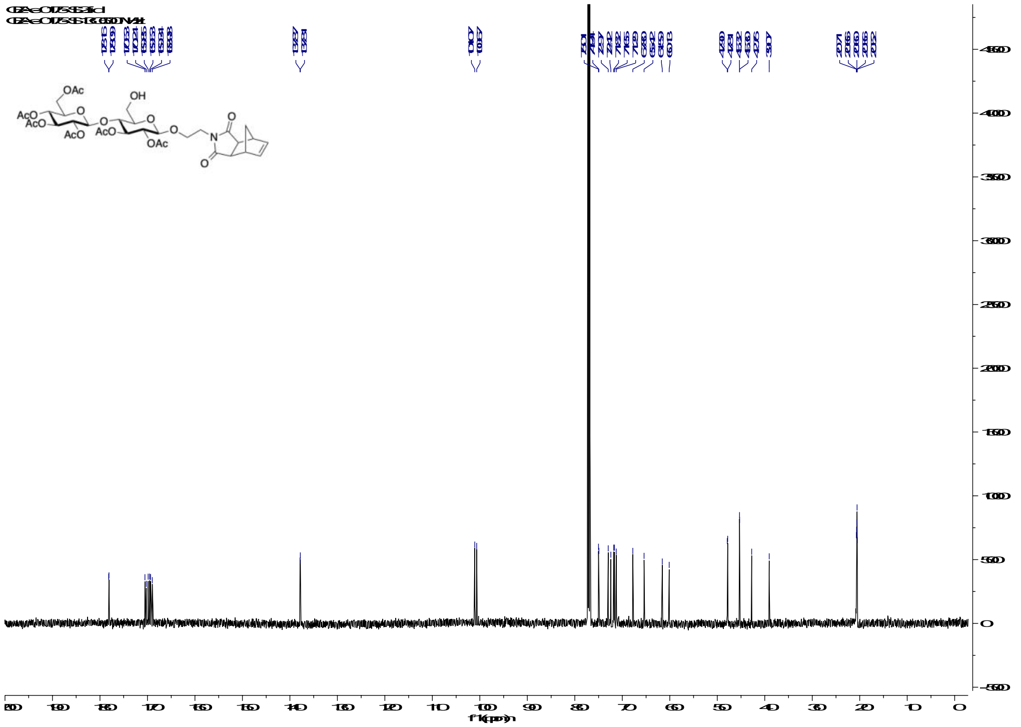

^{1}H NMR of (600 MHz, CDCl3) of Compound 10

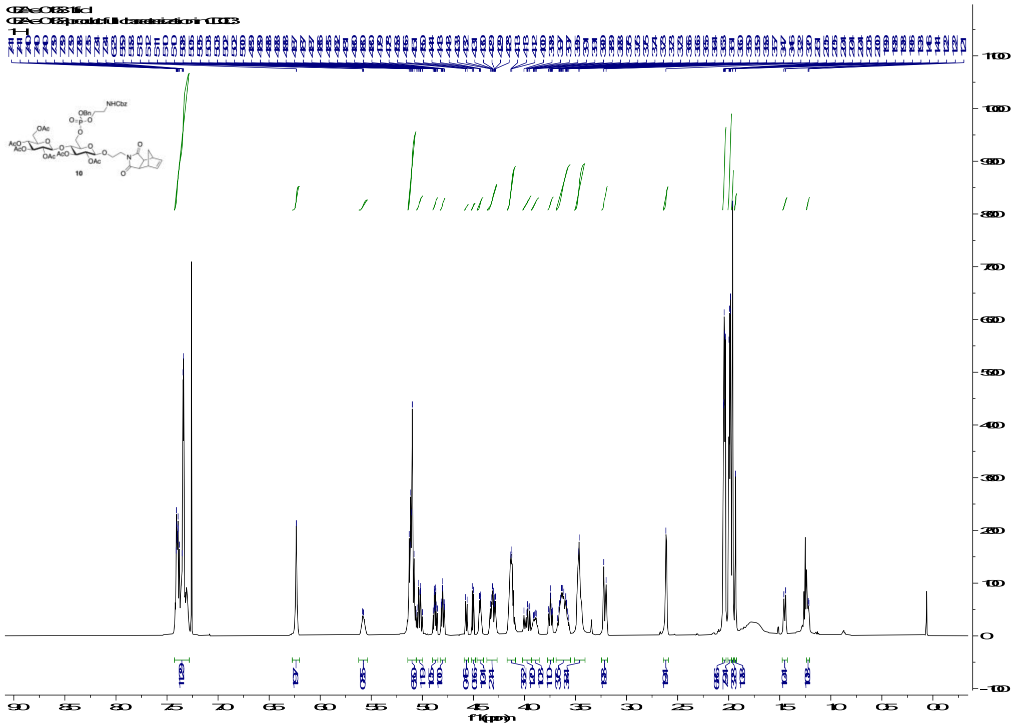

^{13}C NMR of (151 MHz, CDCl3) of Compound 10

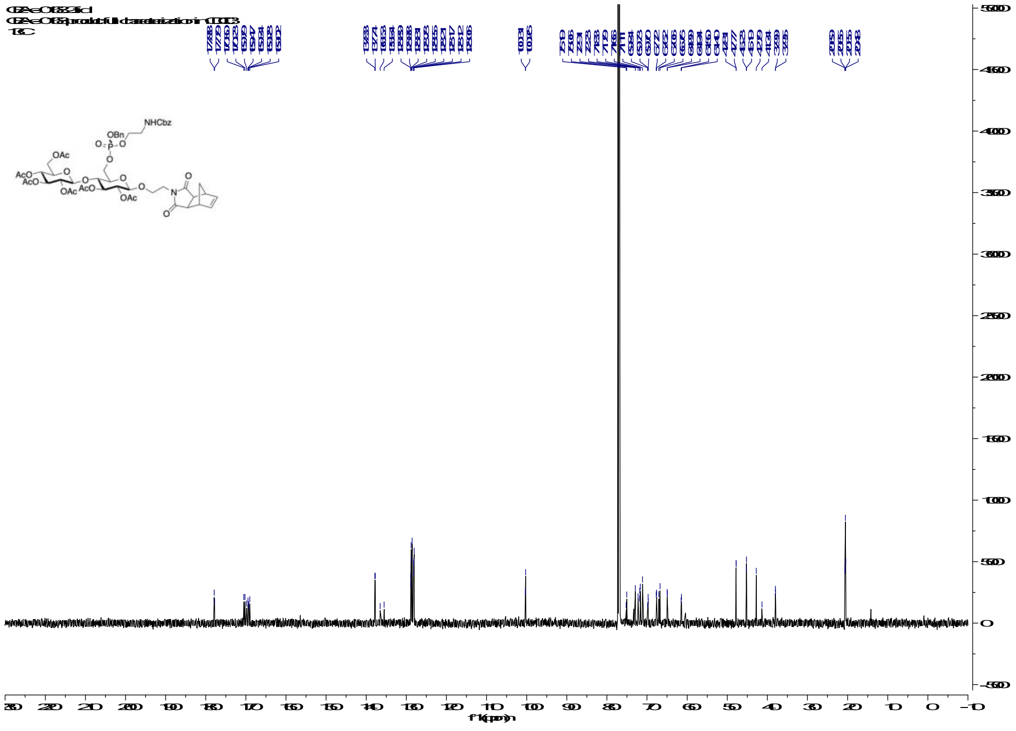

^{31}P NMR of (162 MHz, CDCl3) of Compound 10

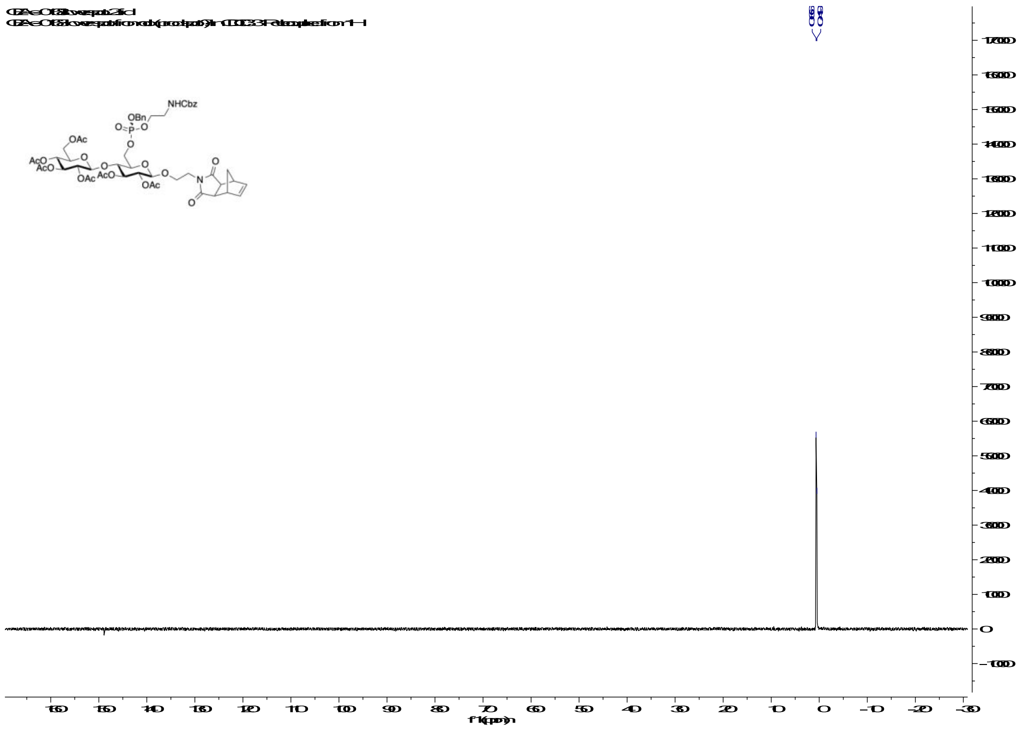

^{1}H NMR of (600 MHz, MeOD) of Compound 11

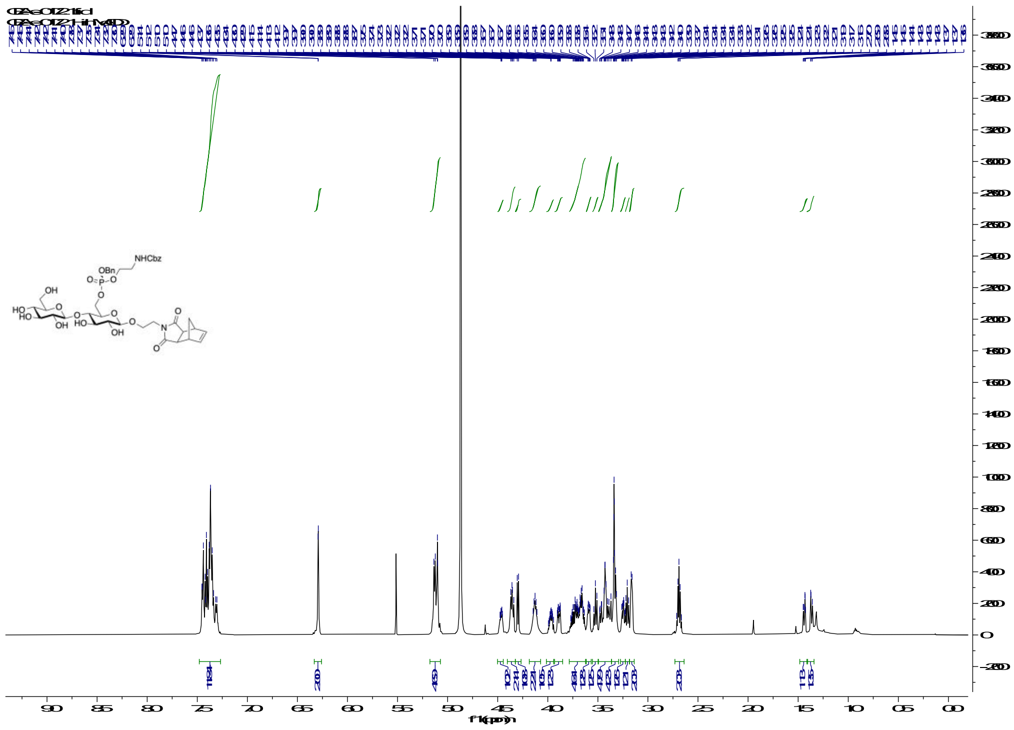

^{13}C NMR of (151 MHz, MeOD) of Compound 11

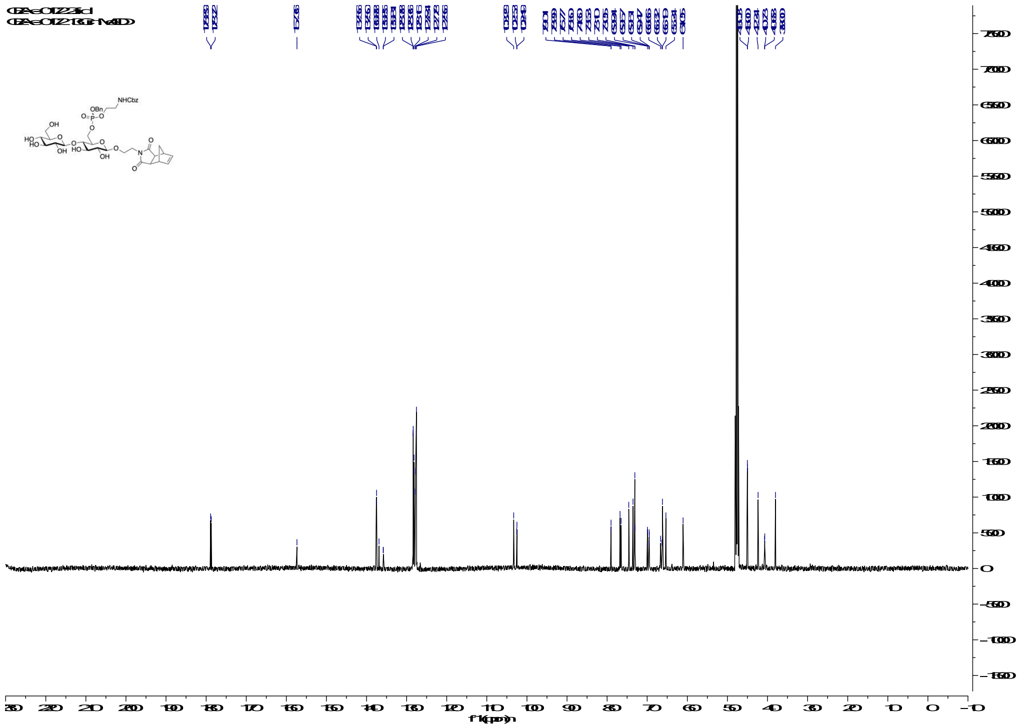

^{1}H NMR of (600 MHz, D_2O) of Glycopolymer 2

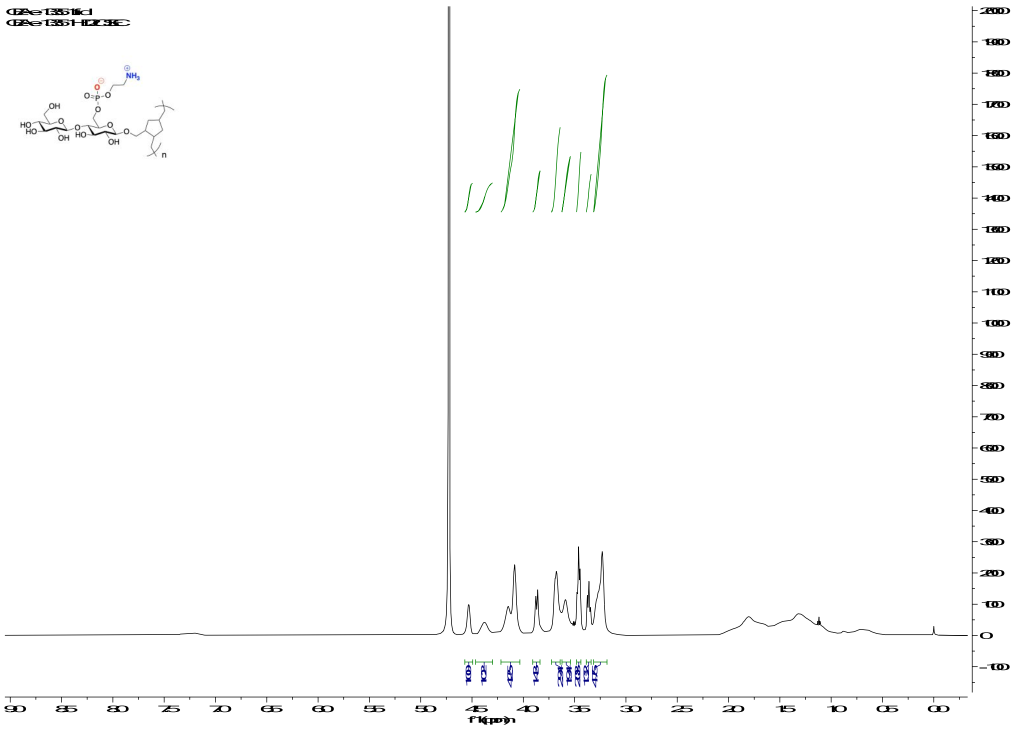

^{31}P NMR of (162 MHz, D_2O) of Glycopolymer 2

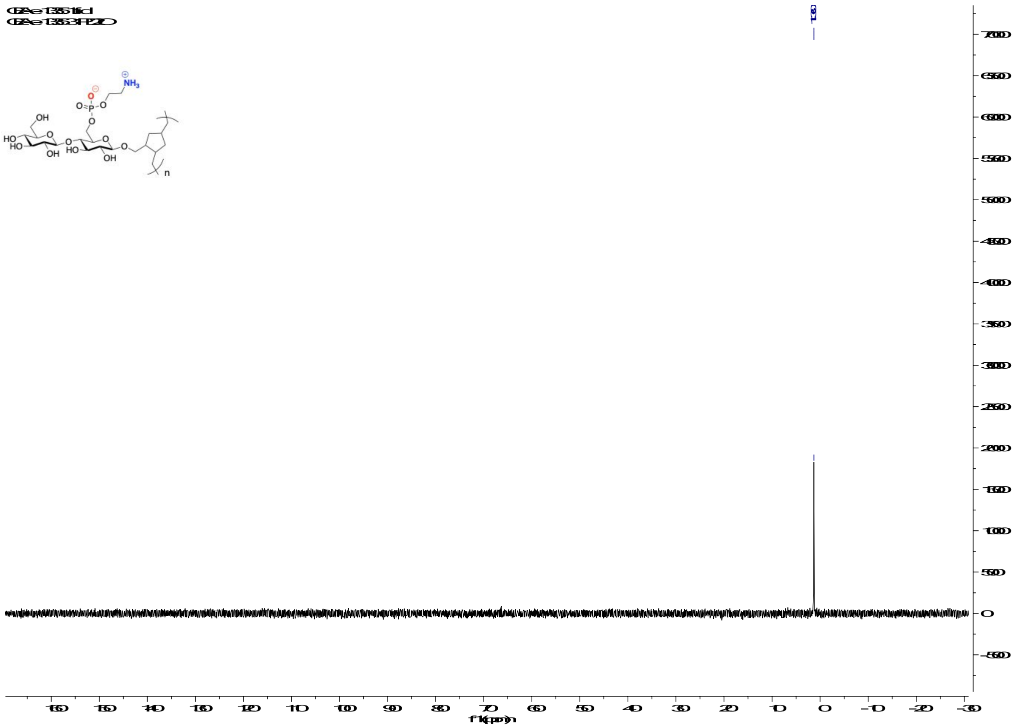

^{1}H NMR of (600 MHz, D_2O) of Glycopolymer 14

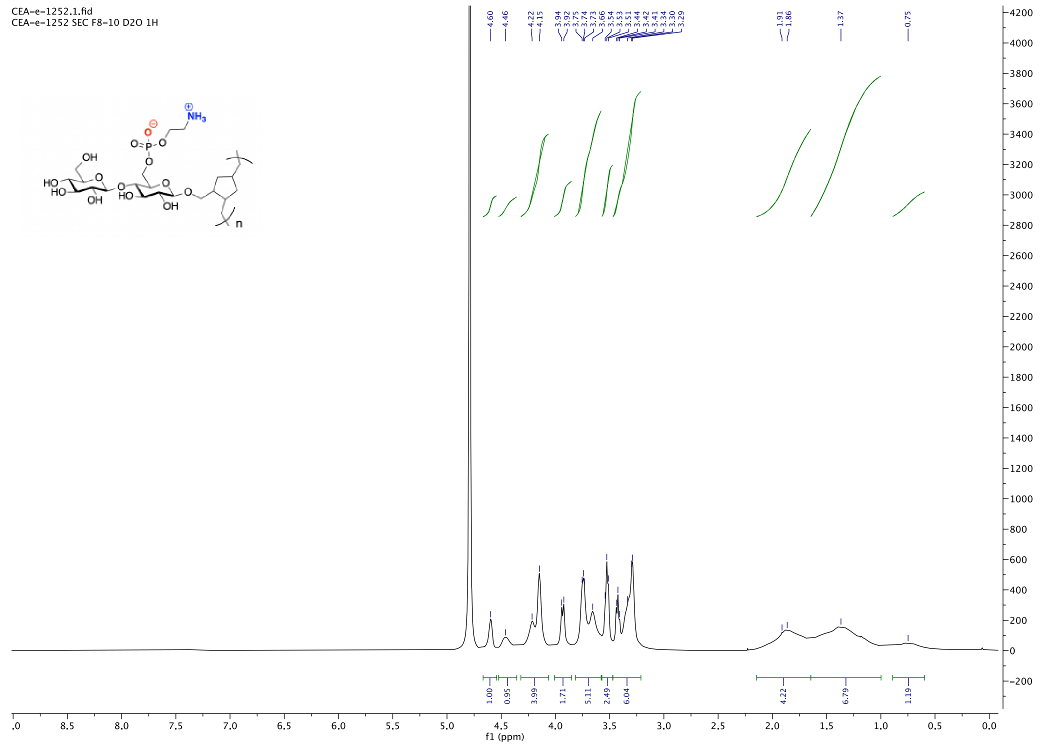

^{31}P NMR of (162 MHz, D_2O) of Glycopolymer 14

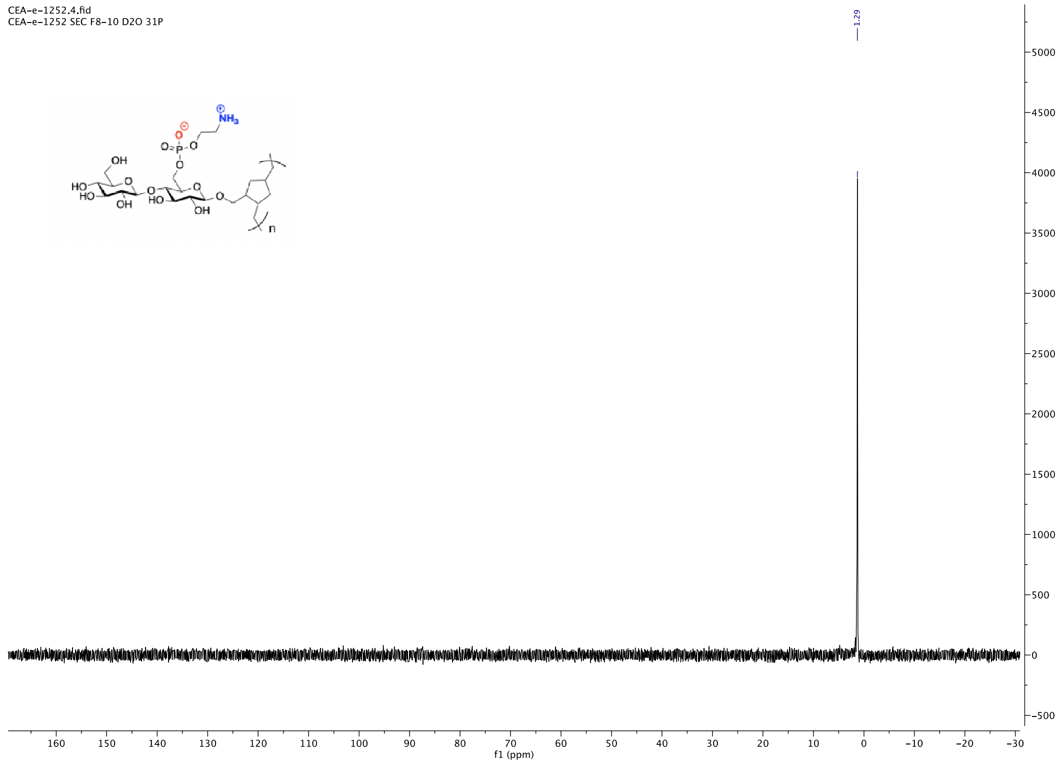

^{1}H NMR of (600 MHz, D_2O) of Glycopolymer 15

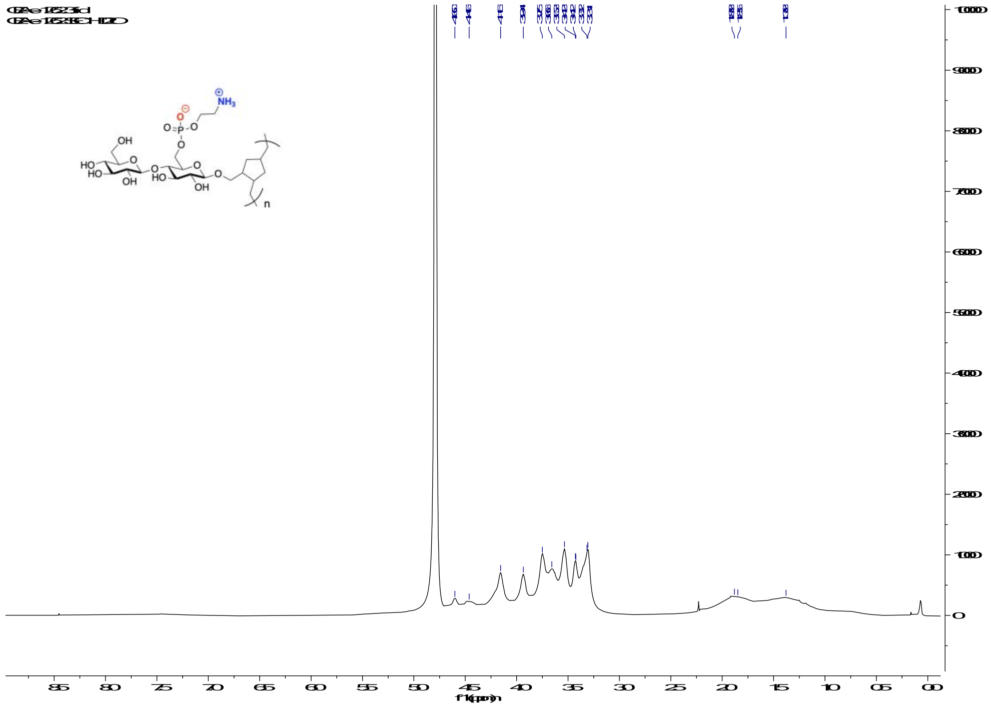

^{31}P NMR of (162 MHz, D_2O) of Glycopolymer 15

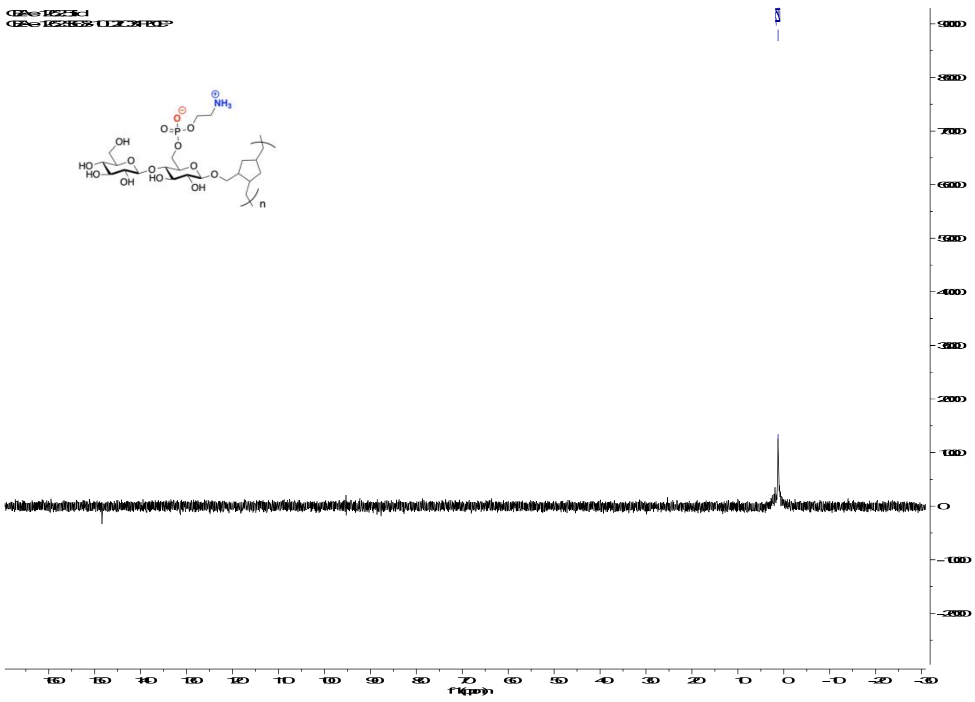

^{1}H NMR of (600 MHz, D_2O) with H_2O pre-saturation of Glycopolymer 16

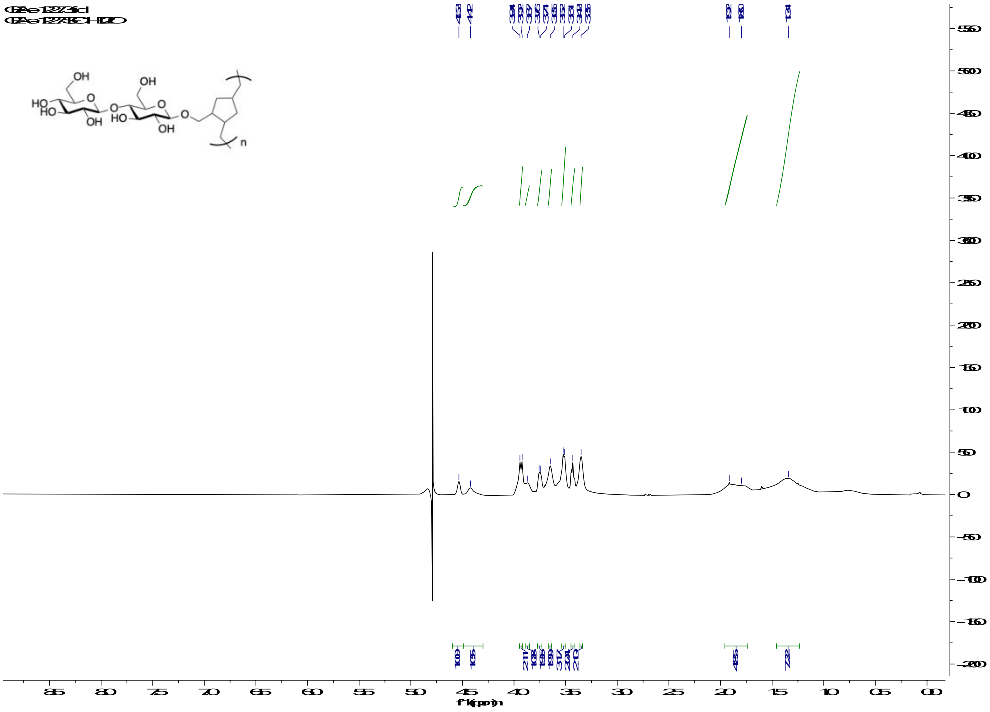

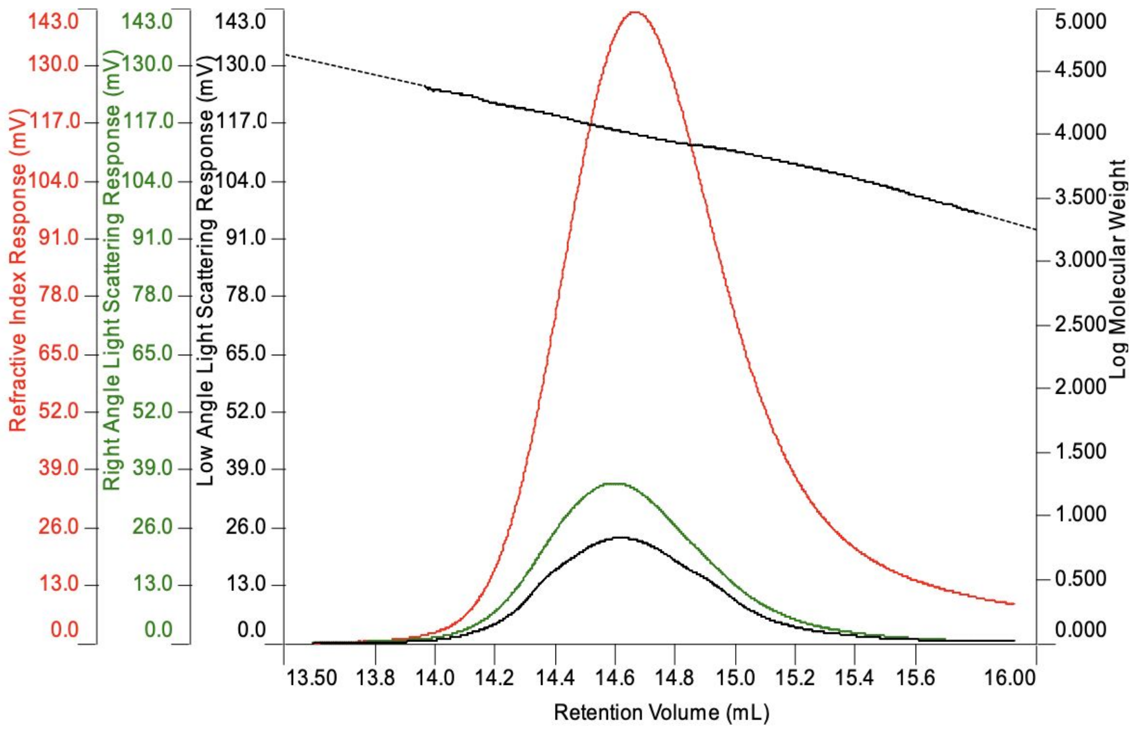

Figure S1. GPC trace of protected n = 8 pEtN cellulose glycopolymer.

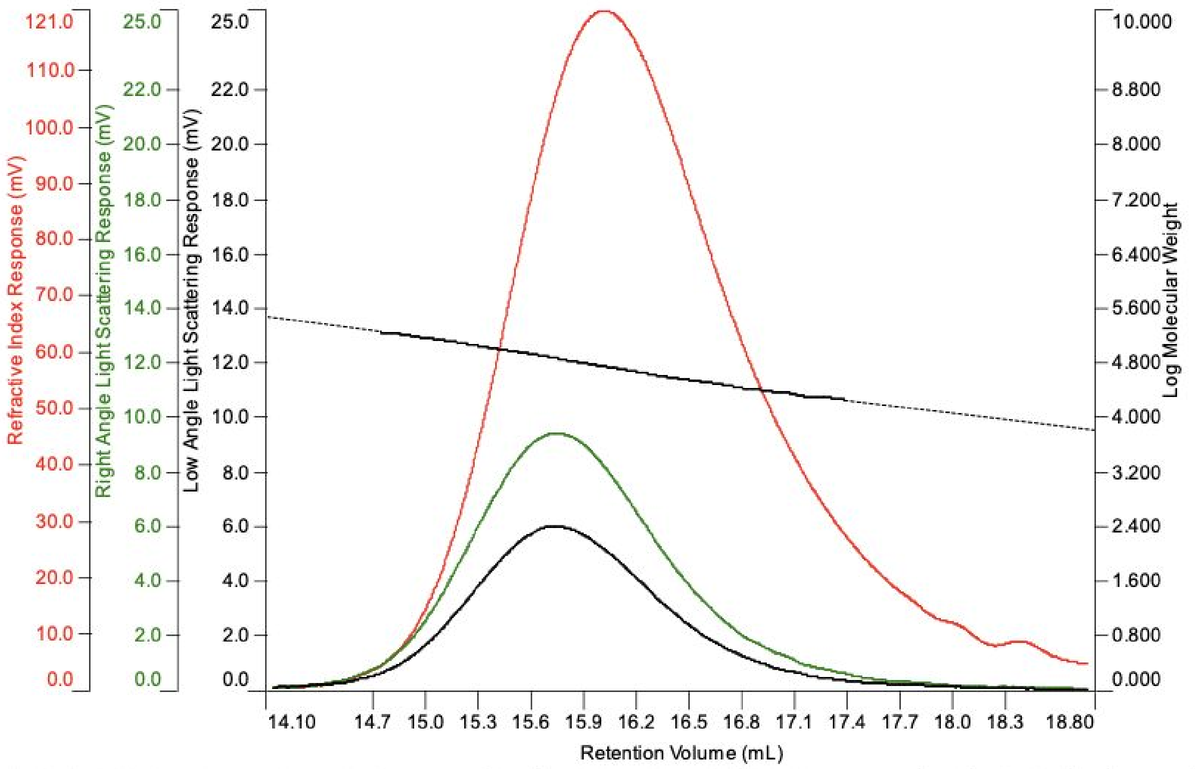

Figure S2. GPC trace of protected n = 34 pEtN cellulose glycopolymer.

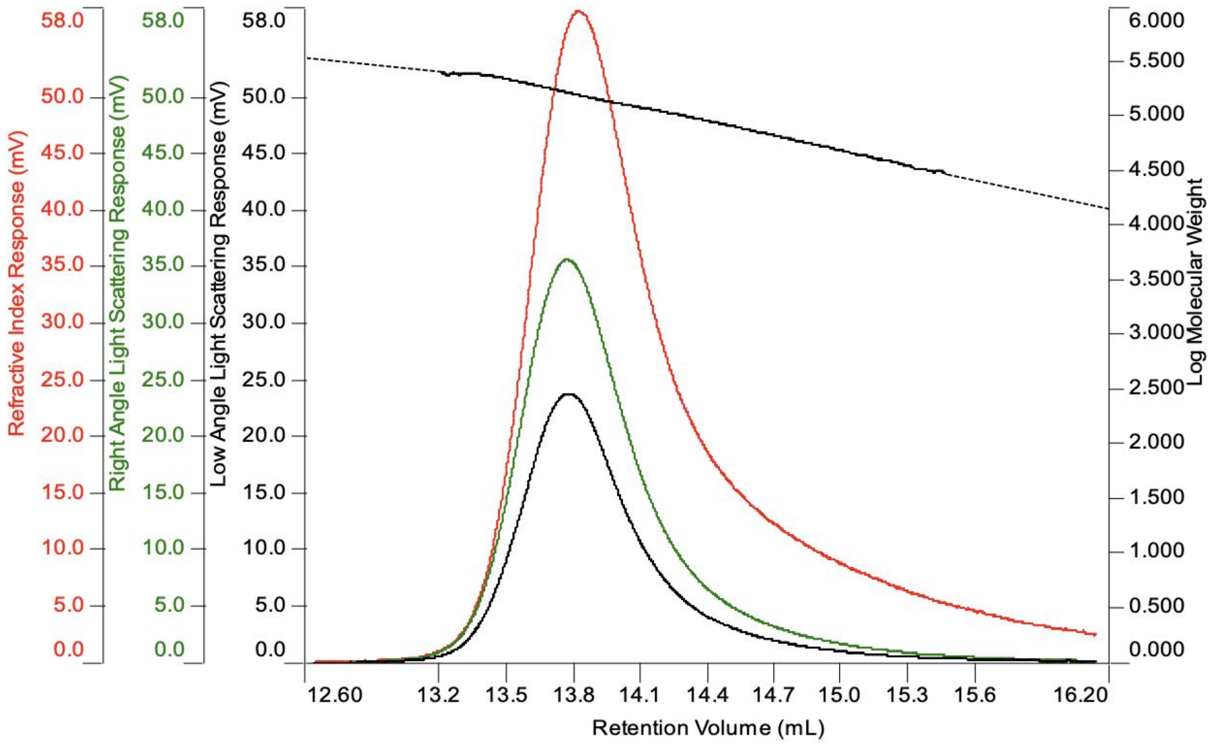

Figure S3. GPC trace of protected n = 82 pEtN cellulose glycopolymer.

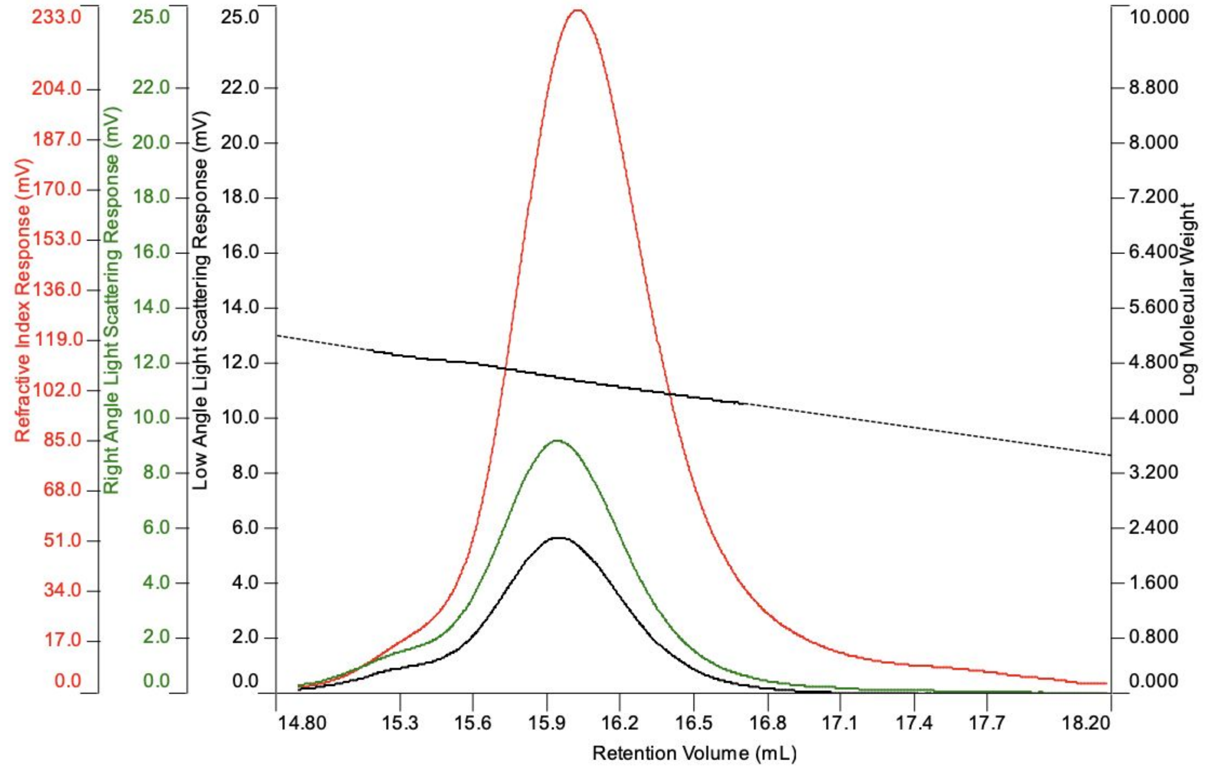

Figure S4. GPC trace of protected n = 36 non-modified cellulose glycopolymer.

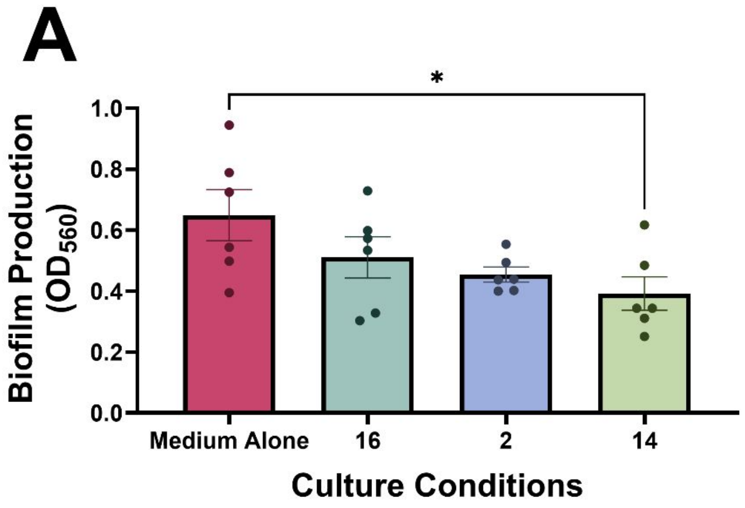

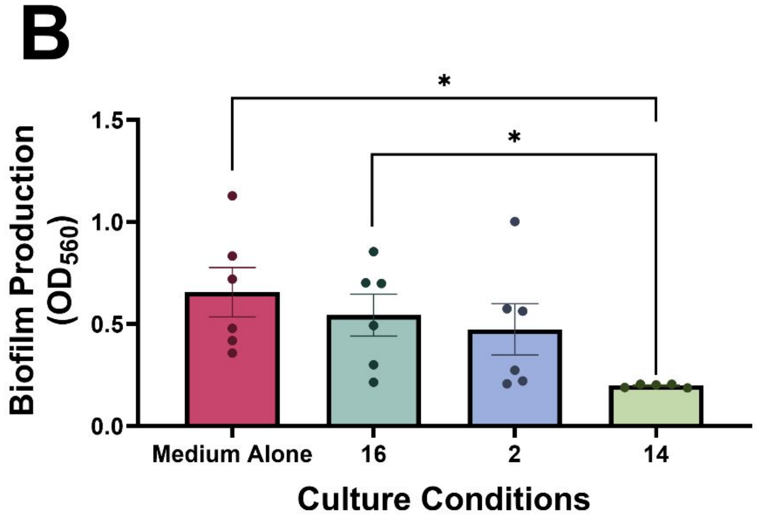

Quantification of bacterial biofilm. Biofilm production was measured by crystal violet staining and spectrophotometric reading at OD_{560} at 24 h post-inoculation. A) Biofilm quantification (OD_{560}) for *E. coli* 11775T in LB is shown. B) Biofilm quantification (OD_{560}) for *E. coli* 700415 in LB is shown. Data displayed represent the relative mean biofilm/biomass ratio ± SEM of at least 3 independent experiments, each with 2 technical replicates. Significant inhibition of biofilm formation was determined by one-way ANOVA with *post hoc* Tukey's test (*$P<0.05$).

References:

1. M. T. Kwasny and G. N. Tew, *J. Mater. Chem. A*. **2017**, *5*, 1400-1405.
2. A. S. Campbell and B. Fraser-Reid, *Bioorganic & Medicinal Chemistry*. **1994**, *2*, 1209-1219
3. R. Xie, I. Lapkriengkri, N. B. Pramanik, S. Mukherjee, J. R. Blankenship, K. Albanese, H. Wang, M. L. Chabinyc, and C. M. Bates. *Macromolecules,* **2002**, *55*, 10513-10521.
4. J. M. Nguyen, R. E. Moore, S. K. Spicer, J. A. Gaddy, S. D. Townsend. *ChemBioChem*, **2021**, *22*, 2540-2545.
